# Secretome Screening of BRAFV600E-Mutated Colon Cancer Cells Resistant to Vemurafenib

**DOI:** 10.3390/biology12040608

**Published:** 2023-04-17

**Authors:** Iris Car, Antje Dittmann, Marko Klobučar, Petra Grbčić, Sandra Kraljević Pavelić, Mirela Sedić

**Affiliations:** 1 Centre for Applied Bioanthropology, Institute for Anthropological Research, Ljudevita Gaja 32, 10000 Zagreb, Croatia; 2 Functional Genomics Center Zurich, ETH Zurich, Winterthurerstr. 190, Y59 H38, 8057 Zurich, Switzerland; 3 Faculty of Medicine, Juraj Dobrila University of Pula, Zagrebačka ul. 30, 52100 Pula, Croatia; 4 Faculty of Health Studies, University of Rijeka, Viktora Cara Emina 5, 51000 Rijeka, Croatia

**Keywords:** BRAFV600E, colon cancer, secretome, BRAF inhibition, vemurafenib, DNA replication, ER stress, RPA1, HSPA5/GRP78, chemoresistance

## Abstract

**Simple Summary:**

Colorectal cancer is the third most common cancer type worldwide. Despite the advancements in pharmacological and surgical treatment approaches, the management of metastatic colon cancer patients carrying BRAFV600E mutation remains challenging due to poor efficacy of chemotherapy drugs. Importantly, targeted therapies were found to induce a complex secretome that stimulates tumor progression and drug resistance. We have accordingly developed an in vitro model of colon cancer cells with BRAFV600E mutation irresponsive to the BRAFV600E inhibitor vemurafenib and analyzed their secretome using two complementary state-of-the-art proteomics technologies. We characterized the cells’ secretome and found proteins implicated in the DNA replication and the endoplasmic reticulum stress to be linked with the development of resistance to vemurafenib. Potential secretome targets for further studies and validation in therapeutic applications include accordingly replication protein A1 and heat shock protein family A member 5.

**Abstract:**

Patients with metastatic colorectal cancer (mCRC) carrying BRAFV600E mutation have worse response to chemotherapy and poor prognosis. The BRAFV600E inhibitor vemurafenib has shown modest efficacy as monotherapy in BRAF-mutated mCRC due to the development of resistance. The aim of this study was to conduct a comparative proteomics profiling of the secretome from vemurafenib-sensitive vs. -resistant colon cancer cells harboring BRAFV600E mutation in order to identify specific secretory features potentially associated with changes in the resistant cells’ phenotype. Towards this aim, we employed two complementary proteomics approaches including two-dimensional gel electrophoresis coupled with MALDI-TOF/TOF mass spectrometry and label-free quantitative LC-MS/MS analysis. Obtained results pointed to aberrant regulation of DNA replication and endoplasmic reticulum stress as the major secretome features associated with chemoresistant phenotype. Accordingly, two proteins implicated in these processes including RPA1 and HSPA5/GRP78 were discussed in more details in the context of biological networks and their importance as potential secretome targets for further functional and clinical evaluation. Expression patterns of RPA1 and HSPA5/GRP78 in tumor tissues from colon cancer patients were also found in additional in silico analyses to be associated with BRAFV600E mutation status, which opens the possibility to extrapolate our findings and their clinical implication to other solid tumors harboring BRAFV600E mutation, such as melanoma.

## 1. Introduction

Approximately 8–12% of patients with metastatic colorectal cancer (mCRC) have BRAFV600E mutation, which is associated with significantly worse response to chemotherapy and poor prognosis [1]. The BRAFV600E inhibitor vemurafenib has shown only modest efficacy as monotherapy in BRAF-mutated mCRC relative to melanoma carrying BRAFV600E mutation due to the development of primary or secondary resistance [2]. In addition, it has been shown that acquired resistance to EGFR/BRAF inhibitors occurs within 4–6 months in individuals who were initially responsive [3]. It has been previously shown that the targeted tumor therapy-induced secretome is involved in phenotypic changes in drug-resistant cancer cell clones that underly metastatic dissemination [4]. Indeed, the cancer cell secretome contains the collection of proteins secreted or shed from cancer cells and has been studied to identify potential novel cancer biomarkers and therapeutic targets [5]. Proteins secreted from cancer cells enter body fluids such as blood or urine, which enables their detection by non-invasive assays [6]. These potentially novel protein biomarkers secreted from cancer cells could aid the identification and the design of novel pharmacological strategies for overcoming the resistance to BRAFV600E inhibition, especially when tumor tissue samples are not readily available.

Preponderant experimental evidence has demonstrated that secreted signals from BRAFV600E-mutated cancer cells play an important role in mediating the response and the development of resistance to targeted therapies. For example, co-culture of vemurafenib-resistant human melanoma cells and sensitive cells treated with vemurafenib significantly augments the growth of resistant cells [4]. Importantly, conditioned media from vemurafenib-sensitive melanoma cells cultured in the presence of vemurafenib facilitate the proliferation of drug-resistant cells [4]. The same study has also revealed that conditioned media from vemurafenib-treated cells exert pro-survival and anti-apoptotic effects in vemurafenib-sensitive melanoma cells treated with vemurafenib. Findings from this study indicate that BRAFV600E mutant melanoma cells respond to BRAF inhibition-induced stress by secreting factors that enable the survival of vemurafenib-sensitive cells and facilitate the growth of vemurafenib-resistant clones. Some of these secreted growth-promoting factors were identified in a similar study in the secretome of BRAF inhibitor-resistant melanoma cells, where endothelin-1 was identified as a molecular factor contributing to paracrine protection of resistant cells from BRAF inhibition [7]. Similarly, a shift of BRAF mutant thyroid cancer cells to more invasive phenotype induced by acquired resistance to Src inhibition was accompanied by changes in the secretome, which again indicates that the secretome composition mirrors phenotypic switching in cancer drug resistance [8].

In this study, we screened the alterations in the secretome accompanying the development of resistance to BRAFV600E inhibition by vemurafenib in BRAFV600E-mutated colon cancer cells. In line with this, we presented data from comparative proteomics profiling of the secretome from vemurafenib-sensitive in comparison with vemurafenib-resistant RKO colon cancer cells harboring BRAFV600E mutation, which were obtained by two complementary proteomics approaches including two-dimensional gel electrophoresis coupled with MALDI-TOF/TOF mass spectrometry and label-free quantitative LC-MS/MS analysis. Gene ontology analysis revealed important groups of secreted proteins within the regulators of DNA replication and the endoplasmic reticulum (ER) homeostasis linked with the unfolded protein response that could be associated with the development of resistance to vemurafenib. Protein–protein interaction (PPI) network analysis and in silico evaluation of selected protein features using the BRAFV600E-mutated colon adenocarcinoma dataset in the Cancer Genome Atlas (TCGA) database pointed to replication protein A1 (RPA1) and heat shock protein family A (Hsp70) member 5 (HSPA5/GRP78) regulating DNA replication and the unfolded protein response, respectively, as potentially interesting protein targets associated with vemurafenib resistance. Further studies are required to investigate the potential of RPA1 and HSPA5/GRP78 in the real-world setting using clinical samples and to examine their roles in determining cancer cell response to BRAFV600E inhibition, not only in colon cancer but also in other BRAFV600E-mutated cancers.

## 2. Materials and Methods

### 2.1. Cell Culturing Conditions and the Development of Vemurafenib-Resistant RKO Colon Cancer Cell Line

The RKO human colon carcinoma cell line harboring BRAFV600E mutation was purchased from the ATCC and maintained in Eagle’s Minimum Essential Medium (Lonza, Switzerland) supplemented with 10% fetal bovine serum (Capricorn Scientific, Ebsdorfergrund, Germany), 2 mM L-glutamine (Capricorn Scientific, Ebsdorfergrund, Germany), penicillin (100 U/mL) (Capricorn Scientific, Ebsdorfergrund, Germany) and streptomycin (100 µg/mL) (Capricorn Scientific, Ebsdorfergrund, Germany) in a humified atmosphere with 5% CO_2_ at 37 °C.

The vemurafenib-resistant RKO cell line was developed by exposing the parental (sensitive) RKO cell line to successively increasing concentrations of Vemurafenib (PLX4032) (MedChemExpress, Monmouth Junction, New York City, NJ, USA) in the period of about 6 months until stable resistance to a clinically relevant concentration of 11.52 µM had been achieved. Established resistance phenotype was confirmed by the MTT assay showing an increase in the IC_50_ value by 10-fold in the resistant RKO cells in comparison with their sensitive counterparts and by microscopic evaluation of the cell morphology as previously reported [9].

### 2.2. Collection of Conditioned Media for the Secretome Analyses

Cells were plated at a seeding density of 4 × 10^6^ into 10 cm Petri dishes in the absence of vemurafenib and grown for 48 h under standard growth conditions. Adhered cells were then washed three times with PBS and two times with EMEM followed by a one-hour incubation in the serum-free medium (SFM). The latter was removed, fresh SFM was added to adherent cells and the cells were grown for the additional 24 h in the absence of vemurafenib in SFM. Conditioned medium was then collected and centrifuged 10 min at 500 rpm and 4 °C followed by second centrifugation at 3000 rpm for 15 min. Adherent cells were detached by trypsin and their viability was examined by the Trypan Blue Exclusion Test. The viability above 95% was the selection criteria for downstream proteomics analyses of conditioned media. Two percent sodium deoxycholate (Sigma-Aldrich, St. Louis, MO, USA) was added to 20 mL of conditioned media to a final concentration of 0.2% sodium deoxycholate followed by incubation on ice for 30 min. Trichloroacetic acid (Sigma-Aldrich, St. Louis, MO, USA) was added to a final concentration of 7.5% (*v*/*v*) and the supernatants were mixed and incubated on ice for 1 h. Proteins were precipitated and centrifuged @ 15,000× *g* for 20 min at 4 °C. Fifty percent of the initial volume of ice-cold (−20 °C) tetrahydrofuran (Sigma-Aldrich, St. Louis, MO, USA) was added to dissolve the pellets and left at −20 °C for 5 min. After centrifugation, 25% of the final volume of ice-cold (−20 °C) tetrahydrofuran was added to the pellets and left at −20 °C for 5 min. After centrifugation, the pellets were air-dried and protein concentration measured by a Qubit™ Protein Assay Kit (ThermoFisher Scientific, Waltham, MA, USA) using a Qubit Fluorometer (ThermoFisher Scientific, Waltham, MA, USA).

### 2.3. Two-Dimensional Gel Electrophoresis (2-DE) and Image Analysis

A total of 300 µg proteins was dissolved in 125 µL 2-DE rehydration buffer containing 7M urea, 2M thiourea, 4% (*w*/*v*) CHAPS, 10% (*w*/*v*) DTT and 0.2% (*w*/*v*) BioLyte^®^ 3/10 Ampholyte (BIO-RAD, Irvine, CA, USA). Dissolved protein samples were loaded onto 7 cm, pH 4–7 ReadyStrip™ IPG Strips (BIO-RAD, Irvine, CA, USA) and the strips were overlaid with mineral oil (BIO-RAD, Irvine, CA, USA). Isoelectric focusing was performed using PROTEAN IEF cell (BIO-RAD, Irvine, CA, USA) under the following conditions: 50 V for 14 h (active rehydration), 250 V rapid for 20 min, 4000 V gradual for 1 h and 4000 V rapid for 15,000 VHours. Proteins were resolved in the second dimension using 12% SDS-polyacrylamide gels at 200 V for 1 h by Mini-PROTEAN Tetra Cell (BIO-RAD, Irvine, CA, USA) followed by gel staining by Coomassie Brilliant Blue G-250 (Sigma-Aldrich, Louis, MO, USA). Gel imaging was performed by ChemiDoc XRS+ Imager (BIO-RAD, Irvine, CA, USA). 2-DE gel image analysis was performed by Progenesis SameSpots 4.0 (TotalLab, Newcastle upon Tyne, Newcastle upon Tyne, UK). The analysis was carried out in triplicate for each condition. Statistically significant differences in protein abundance between the datasets were determined by ANOVA analyses followed by post hoc Tukey’s test.

### 2.4. MALDI-TOF/TOF Mass Spectrometry Analysis

Protein spots of interest were excised from the gel and destained using acetonitrile (Honeywell, Charlotte, NC, USA) and 100 mM ammonium bicarbonate (ABC, Sigma-Aldrich, St. Louis, MO, USA). The samples were then dried in a vacuum concentrator to complete dryness. In-gel tryptic digestion was performed by incubating the gel in 50 mM ABC containing 10 ng/µL trypsin (sequencing grade, Promega, Madison, WI, USA) at 4 °C overnight. Supernatant was collected and the tryptic peptides were extracted by incubation firstly in 65% acetonitrile/5% formic acid solution with shaking and sonication, and then followed by incubation in miliQ water and 100% acetonitrile with shaking and sonication. The collected supernatants were dried in a vacuum concentrator, redissolved in 0.1% trifluoroacetic acid and purified by C18 ZipTip (MerckMillipore, Burlington, MA, USA). The obtained sample was mixed with matrix solution composed of α-cyano-4-hydroxycinnamic acid (0.3 g/L CHCA in the solution containing 2:1 ethanol:acetone, *v*/*v*) at the ratio of 1:10. A volume of 1 µL of the sample/matrix solution mixture was spotted onto the MALDI plate (AnchorChip 800 μm, Bruker Daltonics, Bremen, Germany) and left at room temperature to crystallize. UltrafleXtreme MALDI-TOF/TOF mass spectrometer (Bruker Daltonics, Billerica, MA, USA) was used for MS analyses, where the reflector mode in the m/z range of 700–3500 Da was selected. The MS spectra were externally calibrated using the commercial mixture of Peptide Calibration Standard and Protein Calibration Standard I (Bruker Daltonics, Billerica, MA, USA). FlexControl 3.4 software (Bruker Daltonics, Billerica, MA, USA) was used for acquiring and processing spectra. FlexAnalysis 3.4 (Bruker Daltonics, Billerica, MA, USA) was used for protein database search. Proteins were identified by Mascot 2.4.1 search engine (Matrix Science, London, UK) using the following search parameters: enzyme: trypsin; fixed modifications: carbamidomethylation on cysteine; variable modifications: oxidation on methionine; protein mass: unrestricted; peptide mass tolerance: ±50 ppm; maximum missed cleavage: 2.

### 2.5. LC-MS/MS Sample Preparation

For each conditioned media sample, the volume corresponding to 20 µg of protein was taken and supplemented with 20% sodium dodecyl sulfate (SDS) to a final concentration of 4%. The samples were boiled at 95 °C for 10 min while shaking at 800 rpm on a Thermoshaker (Eppendorf). The samples were reduced with 5 mM dithiothreitol for 30 min at room temperature followed by alkylation with 15 mM iodoacetamide at 50 °C for 30 min in the dark. Samples were processed using the single-pot solid-phase enhanced sample preparation (SP3) [10,11]. In short, protein purification, digestion and peptide clean-up were performed using a KingFisher Flex System (Thermo Fisher Scientific) and carboxylate-modified magnetic particles (GE Life Sciences; GE65152105050250, GE45152105050250) after the manufacturer’s instructions. Resulting digest solution and water elution were combined, dried and re-solubilized in 15 µL of MS sample buffer (3% acetonitrile, 0.1% formic acid).

### 2.6. LC-MS/MS Data Acquisition

Mass spectrometry analysis was carried out using a Q Exactive HF mass spectrometer (Thermo Scientific) equipped with a Digital PicoView source (Waltham, MA, USA) and coupled to a M-Class UPLC (Waters, Milford, MA, USA). Composition of the solvent at the two channels was 0.1% formic acid (channel A) and 0.1% formic acid, 99.9% acetonitrile (channel B). Column temperature was set to 50 °C. For each sample, a total volume of 2 μL was loaded on the ACQUITY UPLC M-Class Symmetry C18 Trap Column (100 Å, 5 µm, 180 µm × 20 mm, Waters) followed by ACQUITY UPLC M-Class HSS T3 Column (100 Å, 1.8 µm, 75 µm × 250 mm, Waters). Peptide elution was performed at a flow rate of 300 nL/min. After a 3 min initial hold at 5% B, a gradient from 5 to 24% B in 80 min and 24 to 36% B in an additional 10 min was applied. The column was cleaned after the run by increasing to 95% B and holding 95% B for 10 min prior to re-establishing loading conditions. Samples were measured in randomized order. The mass spectrometer was operated in data-dependent mode (DDA) on the 12 most abundant ions using Xcalibur (tune version 2.9), with spray voltage set to 2.3 kV, funnel RF level at 60% and heated capillary temperature at 275 °C. Full-scan MS spectra (350−1500 *m/z*) were acquired at a resolution of 120,000 at 200 *m/z* after accumulation to an automated gain control (AGC) target value of 100,000 or for a maximum injection time of 50 ms. Precursors with an intensity above 4500 were selected for MS/MS. Ions were isolated using a quadrupole mass filter with 1.2 *m/z* isolation window and fragmented by higher-energy collisional dissociation (HCD) using a normalized collision energy of 28%. MS2 spectra were recorded at a resolution of 35,000 and a maximum injection time of 54 ms. Charge state screening was enabled, and singly, unassigned charge states and charge states higher than seven were excluded. Precursor masses previously selected for MS/MS measurement were excluded from further selection for 30 s, applying a mass tolerance of 10 ppm. The samples were acquired using internal lock mass calibration on *m/z* 371.1012 and 445.1200. The mass spectrometry data were handled using the local laboratory information management system (LIMS) [12].

### 2.7. LC-MS/MS Data Analysis

Raw MS data were processed using MaxQuant (version 1.6.2.3), and proteins were identified by an integrated Andromeda search engine [13]. Spectra were searched against the Uniprot Homo sapiens reference proteome (taxonomy 9606, canonical version from 9 July 2019), where carbamidomethylation of cysteine was set as fixed modification and methionine oxidation and N-terminal protein acetylation were set as variable. Enzyme specificity was selected to trypsin/P with a minimal peptide length of 7 amino acids and a maximum of two missed cleavages. MaxQuant Orbitrap default search settings were applied. The maximum false discovery rate (FDR) was fixed at 0.01 and 0.05 for peptides and proteins, respectively. Label-free quantification was run and a 2 min window for match between the runs was employed. Individual quantitative values were obtained by the MaxQuant quantitative proteomics software package. Protein fold changes were calculated using the Intensity values from the proteinGroups.txt file. A set of functions in the R package SRMService [14] was applied for selecting proteins with 2 or more peptides, data normalization and calculation of *p*-values (the *t*-test with pooled variance was used). Pseudo fold change was calculated replacing the missing group average by the mean of 10% smallest protein intensities if all measurements of a protein were missing in one of the conditions.

The mass spectrometry proteomics data are deposited to the ProteomeXchange Consortium via the PRIDE [15] partner repository with the dataset identifier PXD039766.

### 2.8. Bioinformatics Analyses

Gene Ontology (GO) enrichment analysis using the DAVID functional annotation tool (https://david.ncifcrf.gov/ accessed on 15 December 2022) [16,17,18,19] was used to elucidate the biological functions of identified proteins, where enriched GO terms with *p* < 0.05 were considered statistically significant.

The Search Tool for Retrieval of Interacting Genes (STRING) (http://string-db.org/ accessed on 15 December 2022) [20] online tool was applied to construct the PPI network, where the confidence score was set to 0.900 (highest confidence). The PPI network was visualized by Cytoscape (https://cytoscape.org/ accessed on 15 December 2022) [21], an open-source software platform for visualizing complex networks. Each node corresponds to a protein, whereas the edges represent the interactions between proteins that contribute to the same biochemical function or pathway. In order to analyze and select significant modules of the PPI network, the Molecular Complex Detection (MCODE) [22] plugin of Cytoscape was employed. To further identify the hub proteins in selected significant modules (clusters), we used Cytohubba [23], a Cytoscape plugin.

In silico evaluation of selected candidate proteins at the mRNA and protein level was conducted using the Cancer Genome Atlas (TCGA) dataset (Colorectal Adenocarcinoma, TCGA, PanCancer Atlas) and the Clinical Proteomic Tumor Analysis Consortium (CPTAC) data, respectively, provided by the cBioPortal for Cancer Genomics (https://www.cbioportal.org/ accessed on 15 December 2022) [24,25]. The total expression of selected proteins in colon cancer was analyzed based on tumor histology using data from the Clinical Proteomic Tumor Analysis Consortium (CPTAC) and the International Cancer Proteogenome Consortium (ICPC) datasets by the means of UALCAN (http://ualcan.path.uab.edu/index.html accessed on 15 December 2022) [26].

## 3. Results

### 3.1. Optimization of Culture Conditions for Secretome Generation

The collection of cell line secretomes from conditioned media for downstream proteomics analyses is challenged by the issue of cell viability, as serum starvation conditions required for secretome collection generally induce cellular stress resulting in apoptotic cell death. Therefore, it is of the outmost importance to optimize cell confluency and incubation time conditions for the recovery of secreted proteins from serum-free conditioned media prior to proteomics analyses to ensure that cell line secretomes reflect the actual cancer biology rather than cell death elicited by culture conditions. For that purpose, parental (vemurafenib-sensitive) RKO colon cancer cells were subjected to serum deprivation for 6, 12, 24, 36, 48 and 72 h, and their cell viability was monitored by Trypan blue exclusion assay. Prominent changes in cell morphology and density could be observed after 48 h of incubation in serum-free medium, while 72 h incubation resulted mainly in rounded cell morphology indicative of apoptosis (Supplementary Figure S1A). Importantly, cell viability peaked at 24 h, reaching 97%, and started to drop from 36 h onwards with a marked decline observed after 72 h (Supplementary Figure S1B). Based on these findings, we selected the 24 h time point as the optimal incubation time for collecting cell secretomes for proteomics analyses, which corresponds well to a previous study in cancer cells which showed that protein secretion kinetics, regardless of the secretion mode, peaks between 24 and 48 h of incubation in serum-free medium [27]. We next investigated the effect of different cell seeding densities on cell viability during the 24 h culturing in serum-free medium and found that cells maintained cell viability by over 95% even at the highest seeding density of 4 × 10^6^ cells (Supplementary Figure S2A), which is considered an acceptable viability for secretome analyses of in vitro cultured cells [28]. Microscopic examination additionally corroborated that cells plated at a density of 4 × 10^6^ did not change their morphology in comparison with those cultured at lower seeding densities (Supplementary Figure S2B), so that this seeding density was chosen as a standard for all subsequent studies.

Switching cultured cells to serum-free media induces cellular stress due to absence of growth factors and other proteins normally present in serum that support cell growth. For this reason, adaptation of cells to serum-free medium is usually recommended, where cells are switched from serum-supplemented medium to serum-free conditions either directly or in several sequential steps. In order to investigate whether the adaptation procedure affects the secretome of RKO cells, we applied two culturing approaches as follows: (1) the cells were directly switched from serum-containing medium into serum-free medium after extensive washing with PBS to remove serum and growth factors and cultured for 24 h; and (2) the cells were subjected to one-hour pre-incubation in serum-free medium, the medium was discarded, cells were extensively washed with PBS and the cells were grown in fresh serum-free medium for 24 h. Cell secretomes were then analyzed by the means of two-dimensional gel electrophoresis (2-DE). As can be seen from Supplementary Figure S3, sequential adaptation produced markedly larger total number of protein spots, especially low-molecular-weight proteins (lower gel region) and was therefore implemented in our methodological framework for the generation of secretomes for all subsequent proteomics analyses.

### 3.2. Proteomics-Based Comparative Analysis of the Secretomes of Sensitive vs. Vemurafenib-Resistant RKO Colon Cancer Cells Carrying BRAFV600E Mutation

After optimization of cell culture conditions for secretome collection, we performed proteomics analyses of secretomes from parental (sensitive) vs. vemurafenib-resistant RKO cells using two complementary proteomics approaches including two-dimensional gel electrophoresis (pH 4–7) coupled to MALDI/TOF-TOF mass spectrometry and label-free quantitative LC-MS/MS. Data from 2-DE/MALDI-TOF/TOF MS analysis revealed 23 differentially expressed proteins (DEPs) with statistical significance (*p* < 0.05), among which 9 and 14 were up- and down-regulated, respectively, in the resistant cell secretome (Supplementary Table S1). Importantly, LC-MS/MS analysis yielded 282 DEPs (*p* < 0.05, log2(FC) > 1), among which 201 and 81 were up- and down-regulated, respectively, in the secretome of resistant cells (Supplementary Table S2). Data obtained from each type of proteomics analysis were combined to generate the lists of up- and down-regulated proteins, which were then subjected individually to bioinformatics analyses for data integration and biological interpretation of up- and down-regulated datasets.

### 3.3. Bioinformatics Analyses of Secretome Data

#### 3.3.1. Upregulated Secretome Dataset

##### Functional and Pathway Enrichment Analysis

**Figure 1:**
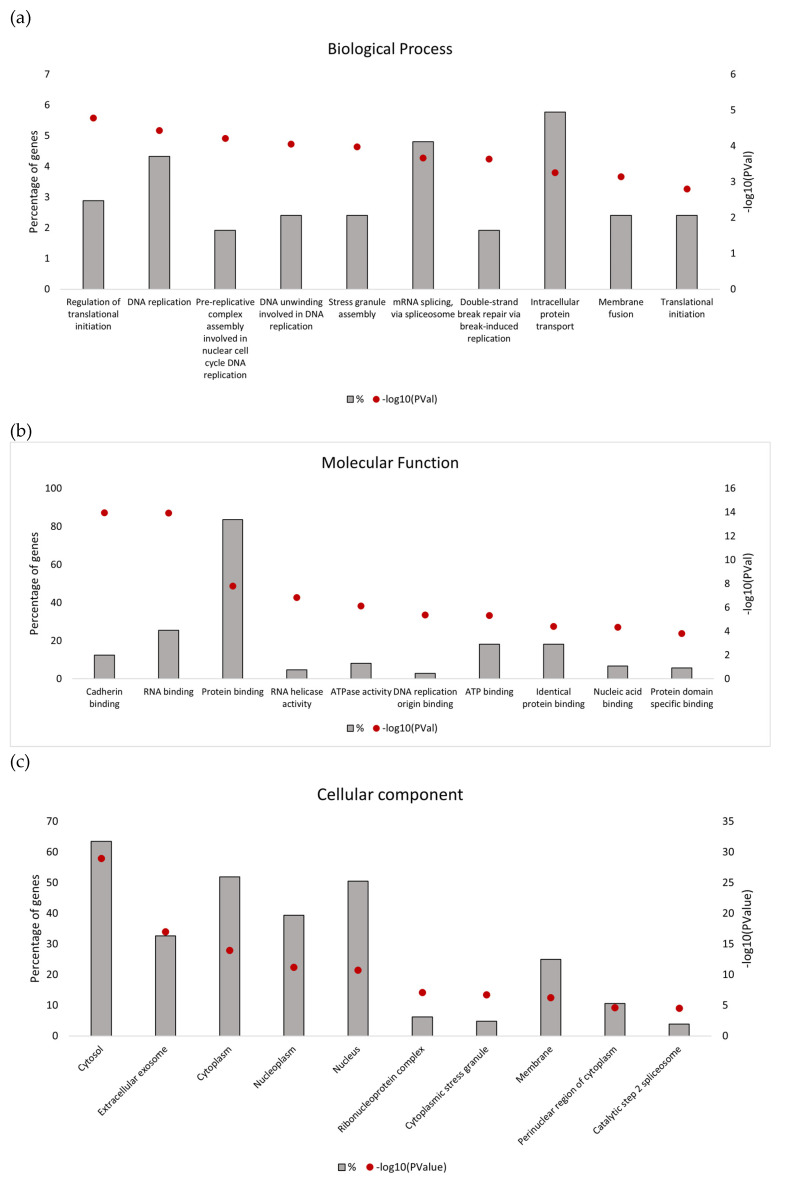
Gene Ontology analysis of upregulated protein dataset associated with vemurafenib resistance in RKO colon cancer cells carrying BRAFV600E mutation. Only those GO terms with *p* < 0.05 were considered statistically significant. Top 10 significant GO terms and pathways are shown. (**a**) Biological process (**b**) Molecular function (**c**) Cellular component.

We performed Gene Ontology (GO) analysis by the Database for Annotation, Visualization and Integrated Discovery (DAVID; https://david.ncifcrf.gov/ accessed on 15 December 2022), where *p* value of <0.05 was set as the cutoff criterion. The top ten GO terms (Figure 1a–c) for upregulated proteins are shown. For biological process (BP), proteins upregulated in vemurafenib-resistant cells were predominantly enriched in the processes related to DNA replication and intracellular protein transport (Figure 1a). Upregulated DEPs in molecular function were significantly associated with protein binding, RNA binding and ATP binding (Figure 1b). The cellular component (CC) analysis indicated that upregulated proteins were mostly located in the cytosol, cytoplasm and nucleus (Figure 1c). This finding is no surprise because previous studies have demonstrated that unconventionally secreted proteins constitute a substantial portion of cancer cell line secretomes and that hundreds of proteins classically located in intracellular organelles/compartments are secreted by tumor cells [27,29]. Altogether, aberrant regulation of DNA replication has emerged as an important upregulated secretory feature linked with vemurafenib resistance in BRAF-mutated colon cancer cells.

###### PPI Network and Module Analysis

The online tool STRING (https://string-db.org/ accessed on 15 December 2022) was used to analyze the protein–protein interaction (PPI) network of upregulated protein dataset with highest confidence interaction score of 0.900, and obtained results were then visualized by Cytoscape software (https://cytoscape.org/ accessed on 15 December 2022 ) (Supplementary Figure S4A–D). The network modular analysis revealed seven highly ranked hub proteins in one significant module including MCM3 (DNA replication licensing factor MCM3), FEN1 (Flap endonuclease 1), MCM5 (DNA replication licensing factor MCM5), MCM2 (DNA Replication Licensing Factor MCM2), MCM6 (DNA Replication Licensing Factor MCM6), PCNA (Proliferating Cell Nuclear Antigen) and RPA1 (Replication protein A 70 kDa DNA-binding subunit). All these hub proteins play a central role in DNA replication and repair.

###### In Silico Evaluation of Selected Upregulated Hub Proteins in BRAFV600E-Mutated Colon Cancer

**Figure 2:**
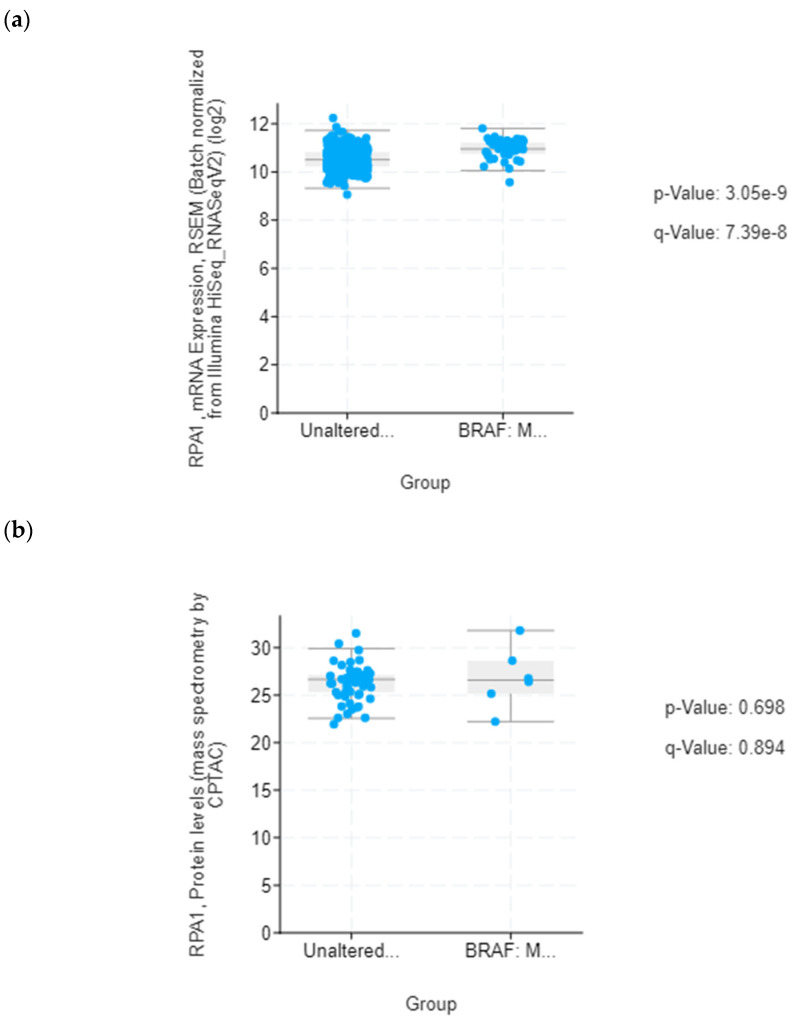

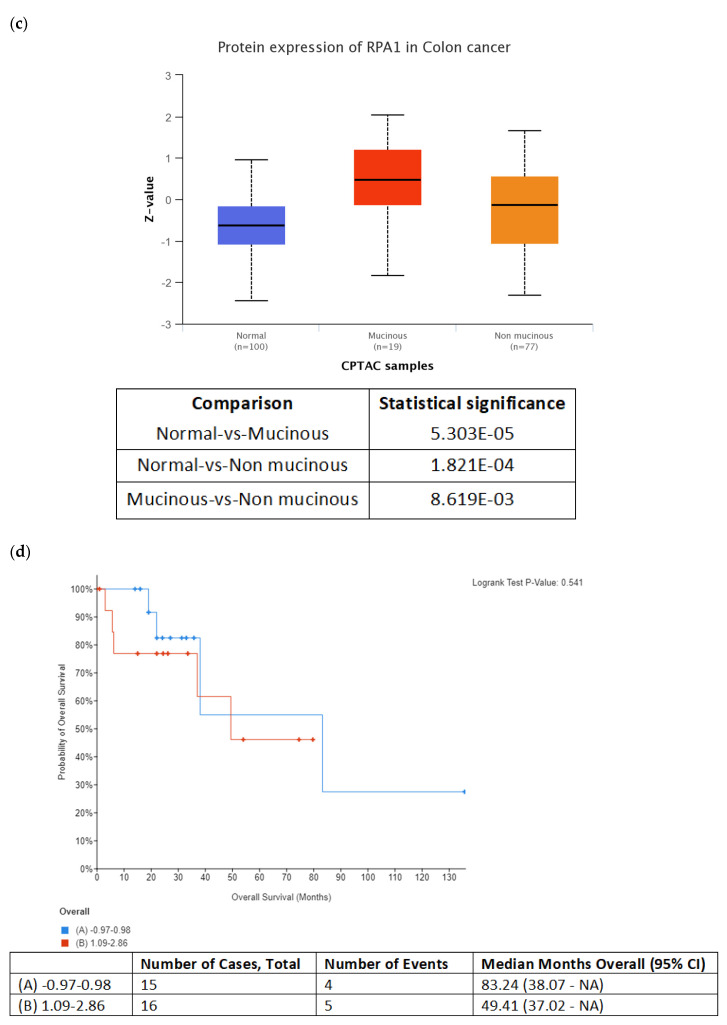
In silico evaluation and survival analysis of selected candidate protein RPA1 (replication protein A1) in BRAFV600E-mutated colon cancer. Evaluation of the RPA1 expression at mRNA (**a**) and protein (**b**) level in the Colorectal Adenocarcinoma (TCGA, PanCancer Atlas) dataset using cBioPortal on-line tool. Expression analysis was conducted in 48 colon cancer samples with BRAFV600E mutation in comparison with 478 samples without BRAF mutation (unaltered group). The RPA1 protein expression was also analyzed in different histological types of colon cancer (normal, mucinous and non-mucinous) in the Clinical Proteomic Tumor Analysis Consortium (CPTAC) dataset using the UALCAN data analysis portal, where Z-values represent standard deviations from the median across samples for the given cancer type (**c**). Median overall survival analysis of RPA1 in 31 cases of colon adenocarcinoma in the TCGA PanCancer Atlas dataset using cBioPortal (**d**). The association between the RPA1 mRNA expression (mRNA expression z-scores relative to all samples (log RNA Seq V2 RSEM)) and the probability of overall survival is shown, where high- and low-mRNA expression groups of colon cancer patients were compared.

Our preliminary results provide a glimpse of the possible cellular processes and the related molecular players that may be implicated in the development of resistance to BRAFV600E inhibition in colon cancer. We next queried if selected hub proteins are specifically associated with BRAFV600E genotype in colon cancer and if their expression changes result from the BRAFV600E-driven biology that affects survival outcome in colon cancer patients. We first explored expression of the genes encoding for the hub proteins identified in our study in the database of tumor tissues from 48 colon cancer patients with BRAFV600E mutation vs. 478 tumor samples without BRAF mutation in the Cancer Genome Atlas (TCGA) dataset (Colorectal Adenocarcinoma, TCGA, PanCancer Atlas) using the cBioPortal for Cancer Genomics (https://www.cbioportal.org/ accessed on 15 December 2022). Among the selected genes, only RPA1, MCM5 and FEN1 had significantly (*p* < 0.05; q < 0.05) higher mRNA expression in BRAFV600E-mutated tumor samples in comparison with unaltered group (Figure 2a, Figures S6A and S7A). Among these three proteins, the analysis showed that only RPA1 had higher protein expression level in BRAFV600E tumor samples in comparison with the unaltered group, albeit without statistical significance (Figure 2b, Figures S6B and S7B). Similar results were obtained by UALCAN (http://ualcan.path.uab.edu/ accessed on 15 December 2022), an interactive web resource for analyzing cancer OMICS data including TCGA and CPTAC and clinical data from 31 cancer types. Proteomic expression profile analysis by UALCAN based on tumor histology using data on colon cancer from the CPTAC dataset revealed that only RPA1 protein expression was significantly increased in mucinous vs. non-mucinous colon cancer (Figure 2c, Figures S6C and S7C). Of note, mucinous histology is a feature of BRAFV600E-mutated colon cancer [1]. Finally, survival analysis by cBioPortal demonstrated that higher mRNA expression of RPA1 correlated with shorter median overall survival of BRAFV600E-mutated patients with colon adenocarcinoma and mucinous adenocarcinoma of the colon (Figure 2d). Altogether, these results indicate a potential oncogenic role of RPA1 in BRAFV600E-mutated colon cancer and suggest that upregulation of RPA1 expression is a molecular feature of BRAFV600E-mutated colon cancer that could be associated with the development of vemurafenib-resistant phenotype. These findings should be further functionally validated, and their translational benefit investigated in a clinical setup.

#### 3.3.2. Downregulated Secretome Dataset

##### Functional and Pathway Enrichment Analysis

**Figure 3:**
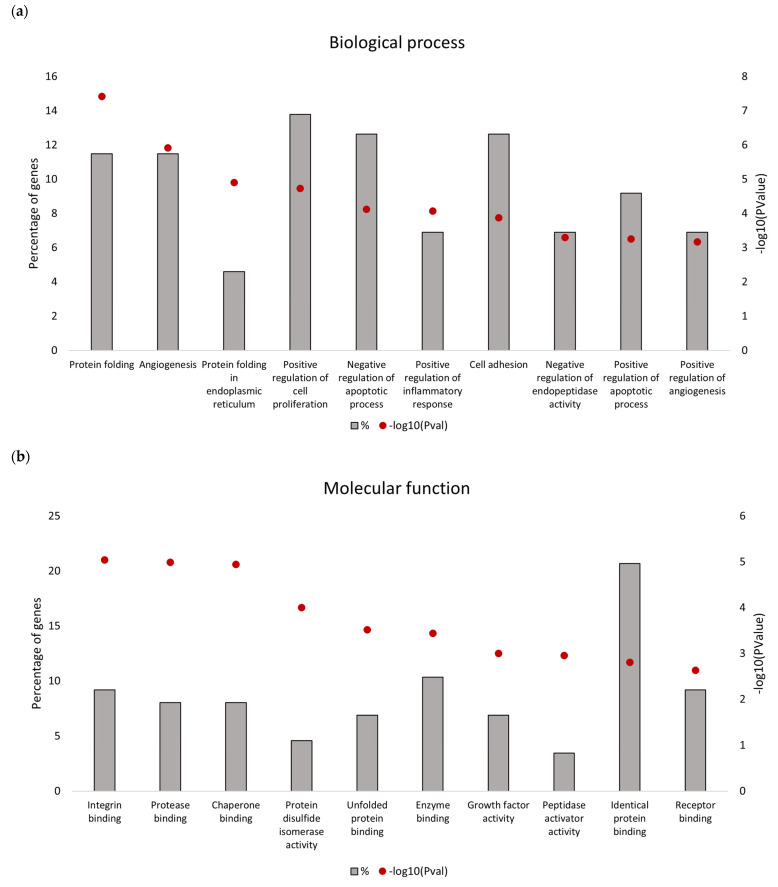

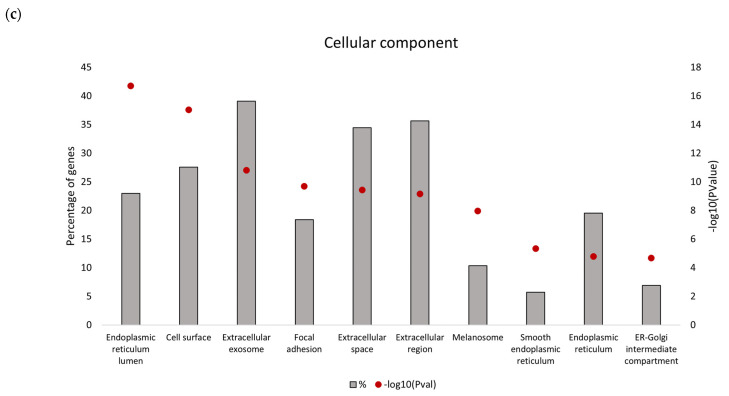
Gene Ontology analysis of downregulated protein dataset associated with vemurafenib resistance in RKO colon cancer cells carrying BRAFV600E mutation. Only those GO terms with *p* < 0.05 were considered statistically significant. Top 10 significant GO terms are shown. (**a**) Biological process (**b**) Molecular function (**c**) Cellular component.

Downregulated proteins were significantly enriched in the biological processes associated with protein folding, angiogenesis, regulation of cell proliferation and apoptosis and cell adhesion (Figure 3a). In the molecular function group, downregulated proteins were mainly enriched in integrin binding, protease binding, chaperone binding, protein disulfide isomerase activity (an endoplasmic reticulum (ER) chaperone that functions in the unfolded protein response), unfolded protein binding, identical protein binding and enzyme binding (Figure 3b). As for the cellular component group, downregulated proteins were mainly localized to the endoplasmic reticulum, cell surface and extracellular space (Figure 3c). These results imply that molecular factors involved in protein folding in the endoplasmic reticulum and the unfolded protein response could represent a prominent downregulated feature of secretory phenotype of vemurafenib-resistant colon cancer cells with BRAFV600E mutation.

##### PPI Network and Module Analysis

Using the STRING database and Cytoscape software, the PPI network of downregulated proteins was constructed (Supplementary Figure S5A). The network module analysis (Supplementary Figure S5B–D) disclosed ten hub proteins in one significant module with five of them being highly ranked, including HSPA5/GRP78 (heat shock 70 kDa protein 5 (glucose-regulated protein, 78 kDa)), HSP90B1 (Heat Shock Protein 90 Beta Family Member 1), PDIA4 (Protein disulfide-isomerase A4), CALR (Calreticulin9 and ITGB1(Integrin beta-1). Apart from ITGB1, a membrane receptor involved in cell adhesion, all other highly ranked hub proteins are localized to the endoplasmic reticulum where they operate as regulators of folding and assembly of proteins and modulators of ER stress and the unfolded protein response (UPR).

##### In Silico Evaluation of Selected Downregulated Hub Proteins in BRAFV600E-Mutated Colon Cancer

**Figure 4:**
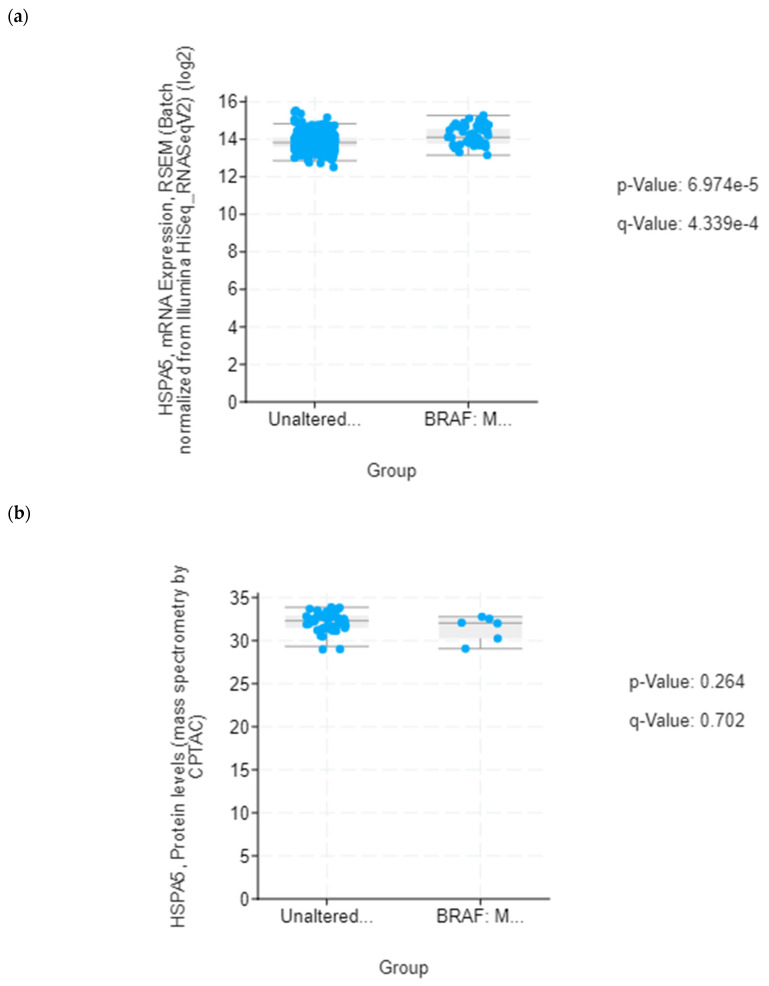

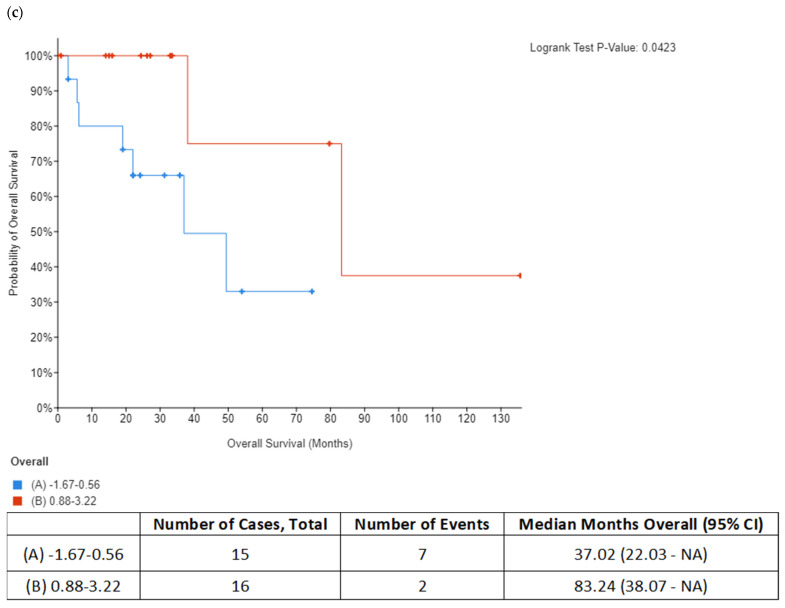
In silico evaluation and survival analysis of selected candidate protein HSPA5/GRP78 (heat shock protein family A (Hsp70) member 5) in BRAFV600E-mutated colon cancer. Evaluation of the HSPA5/GRP78 expression at mRNA (**a**) and protein (**b**) level in the Colorectal Adenocarcinoma (TCGA, PanCancer Atlas) dataset using cBioPortal on-line tool. Expression analysis was conducted in 48 colon cancer samples with BRAFV600E mutation in comparison with 478 samples without BRAF mutation (unaltered group). Median overall survival analysis of HSPA5/GRP78 in 31 cases of colon adenocarcinoma in the TCGA PanCancer Atlas dataset using cBioPortal (**c**). The association between the HSPA5/GRP78 mRNA expression (mRNA expression z-scores relative to all samples (log RNA Seq V2 RSEM)) and the probability of overall survival is shown, where high- and low-mRNA expression groups of colon cancer patients were compared. NA, not available.

Further bioinformatics analyses of the five top-ranked hub proteins revealed their significantly (*p* < 0.05; q < 0.05) increased expression at the mRNA level in tumors from colon cancer patients with BRAFV600E mutation in comparison with an unaltered group (Figure 4a, Figures S8A, S9A, S10A and S11A) with the exception of PDIA4, where statistical significance was not reached. However, the expression level of these five proteins in tumor samples was lower in BRAFV600E-mutated tumors in comparison with an unaltered group, although without statistical significance (Figure 4b, Figures S8B, S9B, S10B and S11B). Importantly, lower mRNA expression of HSPA5/GRP78, HSP90B1, PDIA4, CALR and ITGB1 was associated with poorer median overall survival, especially for HSPA5/GRP78, where this correlation reached statistical significance (Figure 4c, Figures S8C, S9C, S10C and S11C). Thus, decreased level of HSPA5/GRP78 may be investigated in future clinical studies as a clinical endpoint to monitor and/or predict treatment efficacy in BRAFV600E-mutated colon cancer patients.

## 4. Discussion

In the present study, we screened the alterations in the secretome profiles of vemurafenib-sensitive vs. vemurafenib-resistant RKO colon cancer cells carrying BRAFV600E mutation. Although a similar study has already been conducted in BRAFV600E human melanoma cells [30], there are no literature data available on the secretory features concurrent with the development of resistance to BRAFV600E inhibitors in colon cancer. The methodological approach we employed here was based on combining two complementary proteomics platforms including 2-DE/MALDI TOF/TOF MS and LC-MS/MS, which ensured increased proteome coverage and bypassed the limitations inherent to each individual analytical approach. Obtained proteomics datasets were subjected to bioinformatics analyses followed by in silico evaluation of selected up- and down-regulated proteins using the public cancer data repository The Cancer Genome Atlas (TCGA) to examine their expression status specifically in colon cancer patients with BRAFV600E mutation. Such an approach enabled the identification of several promising protein candidates which require further functional and clinical validation to assess their translational value. Biological and cellular functions of these proteins pertinent to the studied cell model will be briefly discussed below.

One of the most relevant upregulated secretome features associated with vemurafenib resistance in BRAF-mutated RKO colon cancer cells identified in this study included DNA replication and repair, where all highly scored hub proteins from the upregulated PPI network play an essential role. Although localized to the nucleus, these proteins were detected in the secretome of colon cancer cells in our study. This finding shows good congruence with a recent study which provided evidence that nuclear proteins regulating DNA replication and repair are enriched in the repertoire of unconventionally secreted proteins from different types of cancer cells [29]. A previous study in breast cancer cells suggested that several nuclear proteins were secreted through exosomes, one of the best characterized routes of non-classical secretory pathways [27], which could also, at least partially, explain why we were able to detect nuclear proteins in cancer cell secretomes. Another explanation why nuclear proteins can be found in cancer cell line secretomes could be provided by the previous findings demonstrating the ability of some secretome proteins classified as nuclear by gene ontology to change their cellular localization to the cytoplasm and extracellular space [27,29].

Previously, the role of DNA replication was demonstrated in the mechanisms governing the resistance of BRAFV600E-mutated colon cancer cells to the MEK inhibitor selumetinib [31]. It was shown that acquisition of MEK inhibitor resistance arose through de novo BRAFV600E amplification resulting from DNA replication during prolonged selumetinib treatment [31]. The same study also revealed that decreasing the frequency of DNA replication during selumetinib treatment suppressed the emergence of resistant clones. Given that MEK is a major downstream mediator of oncogenic BRAF in the MAPK signaling pathway, we cannot exclude the possibility that the same mechanism is involved in the development of resistance to BRAF inhibitor vemurafenib in RKO colon cancer cells harboring BRAFV600E mutation.

The DNA replication licensing system was found to be essential in the progression of cancer. Several DNA replication licensing factors including MCM2, MCM3, MCM5 and MCM6 were found to be significantly upregulated in the secretome from vemurafenib-resistant RKO cells in our study as well. Similarly, prolonged activity of MCM2 was shown to be associated with vemurafenib resistance in melanoma cells [32]. A similar study on BRAFV600E inhibitor-addicted melanoma cells revealed downregulation of MCM2, MCM3 and MCM5 upon drug withdrawal in correlation with reduced cell viability ensuing from discontinued drug treatment [33].

In addition to DNA replication licensing factors, several other proteins involved in the DNA replication process were also found to be increased in the secretome from resistant RKO cells, including FEN1, PCNA and RPA1. However, among all these proteins, RPA1 stands out as the most interesting candidate for future studies as increased RPA1 protein expression in tumor samples from the TCGA colon adenocarcinoma dataset was associated with BRAFV600E genotype and mucinous adenocarcinoma histology characteristic of BRAF mutation tumors. Importantly, our in silico analysis also showed that increased RPA1 expression at the gene level was correlated with poor survival outcomes in BRAFV600E-mutated patients with colon adenocarcinoma and mucinous adenocarcinoma of the colon. Similarly, it was previously reported that RPA1 protein may be used as a prognostic factor in colon cancer patients where increased expression of RPA1 protein was significantly associated with shorter overall survival [34]. Since RPA1 has been shown to act as an oncogene during colorectal cancer development [35], it could potentially serve as diagnostic indicator for colon cancer. Our data encourage further studies to explore the possibility of using circulating RPA1 in monitoring treatment response in BRAFV600E- mutated colon cancer. 

In addition, a previous study on colon cancer cells has shown an association between the levels of RPA1 protein and DNA synthesis rate since the reduction in the in vitro DNA replication activity resulting from tirapazamine (TPZ)-induced inhibition of DNA synthesis was associated with a decrease in the protein level of RPA1 in cytoplasmic extracts of tirapazamine-treated cells [36]. Moreover, the addition of recombinant RPA to the extracts of TPZ-treated cells was able to restore the DNA replication activity to control levels, which suggests that RPA1 mediates the effects of chemotherapeutic agents that inhibit DNA replication. Congruent with these findings, we postulate that increased protein levels of RPA1 detected in the secretome from resistant RKO cells could be potentially indicative of aberrant DNA replication that permits persistent proliferation of resistant cells under prolonged exposure to vemurafenib. Indeed, RPA1 has been previously shown to promote proliferation of colon cancer cells, since knockdown of *RPA1* substantially decreased the rate of cell proliferation, whereas overexpression of *RPA1* substantially raised the cell proliferation rate of colon cancer cells [37]. Importantly, *RPA1* knockdown potentiated the anti-proliferative effect of oxaliplatin in colon cancer cells, which could be ascribed to inhibition of DNA synthesis evidenced by reduced number of cells in the S phase [37]. Altogether, these findings posit that RPA1 could serve as a novel target for chemosensitization in colon cancer. This hypothesis has already been proven in BRAFV600E-mutated colon cancer by showing that knockdown of *RPA1* augments cytotoxic effects of 5-fluorouracil (5-FU) in BRAFV600E-mutated HT-29 colon cancer cells by significantly reducing the proliferative capability of cells measured by the colony formation assay and enhancing 5-FU-induced apoptosis [35].

In addition to mediating the effects of chemotherapy, protein machinery involved in DNA replication plays an important role as downstream effectors of cytotoxic effects of radiation in BRAF-mutated colon cancer cells. For example, radiation induces a dose-dependent increase in the chromatin-bound RPA70 (Replication protein A 70 kDa DNA-binding subunit) protein content in RKO cells [38]. Importantly, decreased level of the KIN17 protein implicated in DNA replication improves radiosensitivity in RKO cells. Thus, the concept based on interfering with the DNA replication proteins could prove beneficial to improving the effectiveness of chemotherapy in treating BRAFV600E-mutated colon cancer. In this context, targeting RPA1 may represent a novel and promising strategy to overcome resistance to BRAFV600E inhibition in colon cancer. This possibility should be additionally explored in future studies.

Downregulated proteins identified in the secretome from resistant RKO cells were mostly related to the endoplasmic reticulum (ER) function associated with protein folding and processing in the endoplasmic reticulum and the unfolded protein response (UPR). Bioinformatics analysis identified that the five most significant hub proteins in the downregulated dataset are involved in ER stress and the unfolded protein response (HSPA5/GRP78, HSP90B1, PDIA4 and CALR) and cell adhesion (ITGB1). Thus, aberrant regulation of the ER function could be associated with the BRAFV600E genotype in colon cancer. Indeed, previous findings have demonstrated that oncogenic BRAF induces chronic ER stress in BRAF-mutated colon cancer cells [39]. In addition, the UPR was previously identified as a pathway significantly associated with the BRAF-mutated colorectal cancer subgroup with poor prognosis [39]. Although lower mRNA expression levels of several UPR-related proteins identified in this study were found in association with poorer median overall survival of BRAF-mutated colon cancer patients, it was only for HSPA5/GRP78 that this correlation was statistically significant. Based on this in silico finding, we propose that reduced level of secreted HSPA5/GRP78 should be further investigated as a potential indicator of treatment outcomes in BRAF-mutated colon cancer.

HSPA5 or Glucose-Regulated Protein 78 kDa (GRP78) is a pivotal regulator of the unfolded protein response and the apoptotic machinery linked with the ER [40]. HSPA5/GRP78 regulates the UPR by binding to and inactivating ER stress sensors under non-stressed conditions. When misfolded proteins accumulate in the ER leading to ER stress, HSPA5/GRP78 binds to misfolded proteins, releases the UPR sensors and triggers the UPR. Heijmans et al. [41] reported that depletion or inactivation of HSPA5/GRP78 induced the UPR in LS174T colorectal cancer cells, and suggested that depletion of HSPA5/GRP78 can serve as a bona fide model to study ER stress signaling. Although HSPA5/GRP78 is mostly localized to the ER lumen, ER stress promotes the translocation of a subfraction of HSPA5/GRP78 to the cell surface and its secretion [40]. Based on these studies, we hypothesize that decreased expression of HSPA5/GRP78 secreted in the conditioned medium from vemurafenib-resistant RKO cells could be indicative of activation of the UPR resulting from ER stress under long-term vemurafenib exposure. Previously, it was demonstrated that HSPA5/GRP78 inhibition evoked ER stress and apoptosis in a panel of BRAFV600E-mutated colon cancer cell lines [39]. Similarly, downregulation of the HSPA5/GRP78 protein expression reduced the viability of BRAF mutant colon cancer cells [39,42], which indicates that BRAF-mutated colon cancer cells are dependent on the UPR for survival. Knowing that UPR activation resulting from continuous exposure to mild ER stress allows cell survival [39,42,43], we propose that downregulation of HSPA5/GRP78 in resistant RKO cells could be a part of adaptive response to chronic stress induced by prolonged exposure to a low concentration (11.52 µM) of vemurafenib, conferring a growth advantage to resistant cells. Further studies are required to corroborate the involvement of ER stress and the UPR in the development of resistance to vemurafenib and to elucidate the role of circulating HSPA5/GRP78 in drug resistance mechanisms in BRAFV600E-mutated colon cancer.

In summary, increased level of secreted RPA1 protein was identified in this study as a prominent secretory feature of vemurafenib-resistant phenotype in BRAFV600E mutant colon cancer cells and detected in additional in silico analyses as a molecular trait of tumors from BRAFV600E-mutated colon cancer patients associated with unfavorable survival outcome. Thus, upregulation of secreted RPA1 protein could represent an important prognostic factor and a potential novel target for chemosensitization in BRAFV600E-mutated colon cancer. Likewise, down-regulation of secretory HSPA5/GRP78 protein emerged in our study as a significant feature associated with chemoresistant phenotype, which was also found in in silico analyses to be correlated with BRAFV600E genotype and poor survival rates in colon cancer patients. These findings warrant further validation in clinical samples, which are currently limited by a small number of metastatic colon cancer patients receiving therapy with BRAFV600E inhibitors in national clinical centers that could provide further clinical support to our findings due to the relatively low incidence of BRAFV600E mutation among the colon cancer patient population and high costs of the therapy. To address these challenges, multi-centric validation studies will be required.

Given that the mechanisms underlying primary resistance to BRAFV600E inhibitors in BRAF-mutated metastatic colon cancer are not fully elucidated due to heterogeneity in drug response derived from molecular heterogeneity of BRAFV600E-mutated colorectal cancers [44] and knowing that no currently established biomarkers predict primary resistance to anti-BRAF therapy in colon cancer [45], our findings could lay the groundwork for future studies to identify clinically relevant blood protein candidates with a predictive value that could guide the clinical management of colon cancer patients on anti-BRAF therapy. This is particularly important for patients with surgically unresectable disease and aggressive disease with metastases where tumor tissue is not readily available; circulatory markers, such as RPA1 and HSPA5/GRP78 proteins, may represent an informative tool to monitor the response to BRAF inhibitors and navigate clinical decision-making.

## 5. Conclusions

The major secretome features associated with acquired resistance to vemurafenib in BRAF-mutated colon cancer cells revealed in this study included deregulation of DNA replication and ER function. From the translational point of view, the most relevant secretory proteins associated with acquisition of chemoresistant phenotype were RPA1 and HSPA5/GRP78, implicated in the regulation of DNA replication and induction of the unfolded protein response, respectively. Increased level of RPA1 and reduced level of HSPA5/GRP78 were the most prominent secretory features of vemurafenib-resistant colon cancer cells, which were also specifically correlated with BRAFV600E genotype and poor survival outcomes in colon cancer patients with BRAFV600E mutation. These findings should be, however, functionally validated and their prognostic potentials further explored in future clinical studies in BRAFV600E-mutated colon cancer patients. Since the expression pattern of RPA1 and HSPA5/GRP78 in tumor tissues from colon cancer patients was found in in silico analyses to be associated with BRAFV600E mutation status, the possibility to extrapolate our findings and their clinical implication to other solid tumors harboring BRAFV600E mutation, such as melanoma, should also be further explored.

## Data Availability

The mass spectrometry proteomics data have been deposited to the ProteomeXchange Consortium via the PRIDE [15] partner repository with the dataset identifier PXD039766.

## Supplementary Materials

The following supporting information can be downloaded at https://www.mdpi.com/article/10.3390/biology12040608/s1: Figure S1: Optimization of incubation time for sensitive RKO colon cancer cells culturing under the serum starvation conditions for the collection of secretome prior to downstream proteomics analyses; Figure S2: Optimization of the sensitive RKO cell seeding density for the collection of secretome prior to downstream proteomics analyses; Figure S3: The effect of adaptation procedure to serum-free growth conditions on the secretome quality of the sensitive RKO cells; Figure S4: Construction and modular analysis of the PPI network of upregulated proteins and identification of hub proteins; Figure S5: Construction and modular analysis of the PPI network of downregulated proteins and identification of hub proteins; Figure S6: In silico validation and survival analysis of selected candidate protein MCM5 in BRAFV600E mutated colon cancer; Figure S7: In silico validation and survival analysis of selected candidate protein FEN1 in BRAFV600E mutated colon cancer; Figure S8: In silico validation and survival analysis of selected candidate protein HSP90B1 in BRAFV600E mutated colon cancer; Figure S9: In silico validation and survival analysis of selected candidate protein PDIA4 in BRAFV600E mutated colon cancer; Figure S10: In silico validation and survival analysis of selected candidate protein CALR in BRAFV600E mutated colon cancer; Figure S11: In silico validation and survival analysis of selected candidate protein ITGB1 in BRAFV600E mutated colon cancer; Table S1: List of differentially expressed proteins with statistical significance (*p* < 0.05) in the secretome of vemurafenib-resistant in comparison with vemurafenib-sensitive RKO colon cancer cells identified by 2-DE coupled with MALDI-TOF/TOF mass spectrometry analysis; Table S2: List of differentially expressed proteins with statistical significance (*p* < 0.05, log2(FC) > 1) in the secretome of vemurafenib-resistant in comparison with vemurafenib-sensitive RKO colon cancer cells revealed by label-free quantitative LC-MS/MS analysis.
