# Supplementary material for: Secretome Screening of BRAFV600E-Mutated Colon Cancer Cells Resistant to Vemurafenib
Source: Biology (Basel). 2023 Apr 17;12(4):608. doi: 10.3390/biology12040608 (PMC10136293; doi:10.3390/biology12040608)
Supplement: Supplementary file 1 [file biology-12-00608-s001.zip › biology-2229854-supplementary.pdf]

Supplementary Material

Secretome screening of BRAFV600E mutated colon cancer cells resistant to vemurafenib

Iris Car [1], Antje Dittmann [2], Marko Klobučar [1] **, Petra Grbčić [3] **, Sandra Kraljević Pavelić [4] * and Mirela Sedić [1] *

[1] Centre for Applied Bioanthropology, Institute for Anthropological Research, Ljudevita Gaja 32, 10000 Zagreb, Croatia

[2] ETH Zurich, Functional Genomics Center Zurich, Winterthurerstr. 190, Y59 H38, 8057, Zurich, Switzerland

[3] Juraj Dobrila University of Pula, Zagrebačka ul. 30, 52100 Pula, Croatia

[4] University of Rijeka Faculty of Health Studies, Viktora Cara Emina 5, 51000 Rijeka, Croatia

* **Correspondence**: mirela.sedic@inantro.hr; Tel.: +385 1 5535135 (MS); sandrakp@uniri.hr; Tel.: + 385 51 554 947 (SKP)

**** Equal contribution**

Supplementary Materials: The following supporting information can be downloaded at: https://www.mdpi.com/article/10.3390/biology12040608/s1

Contents

1. *In vitro* model of BRAFV600E mutated colon cancer cells 3

2. PPI network and module analysis 6

2.2. Construction and modular analysis of the PPI network of downregulated proteins and identification of hub proteins 8

3. *In silico* evaluation of selected PPI network hub proteins in BRAF V600E-mutated colon cancer 10

***3.1. In silico* evaluation of selected hub proteins from the upregulated PPI network in BRAF V600E mutated colon cancer** 11

***3.2. In silico* evaluation of selected hub proteins from the downregulated PPI network in BRAF V600E mutated colon cancer** 15

1. *In vitro* model of BRAFV600E mutated colon cancer cells

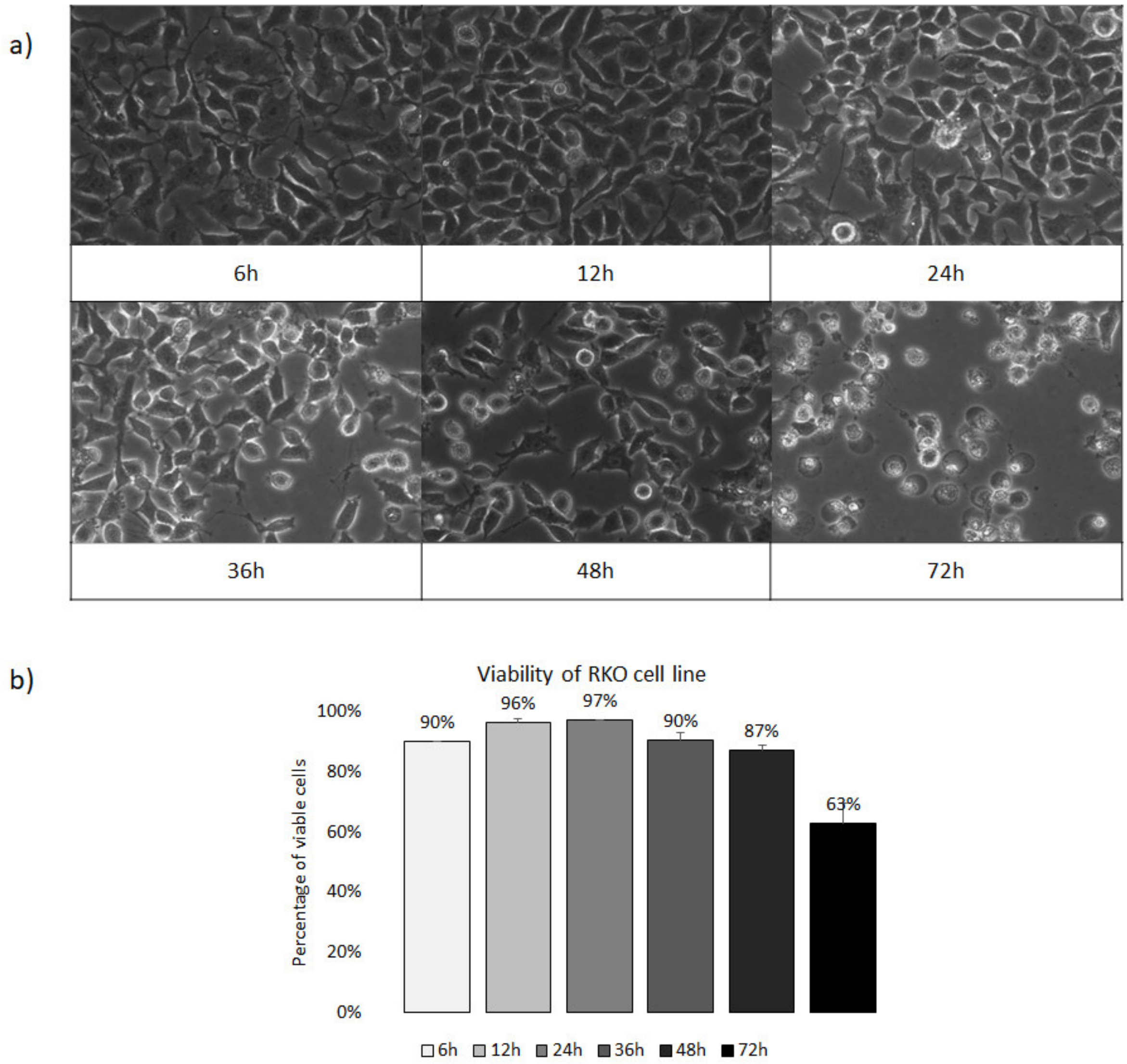

Figure S1: Optimization of incubation time for sensitive RKO colon cancer cells culturing under the serum starvation conditions for the collection of secretome prior to downstream proteomics analyses. The cells were plated at a seeding density of 1 x 106 onto a 10-cm Petri dish, and their morphology was examined by light microscopy (10x magnification) at indicated time points (A). Cell viability was measured by the Trypan blue exclusion assay to investigate the effect of different incubation times on cell survival (B).

a)

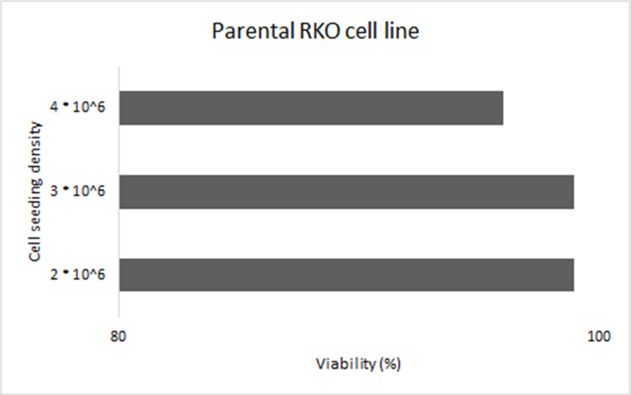

b)

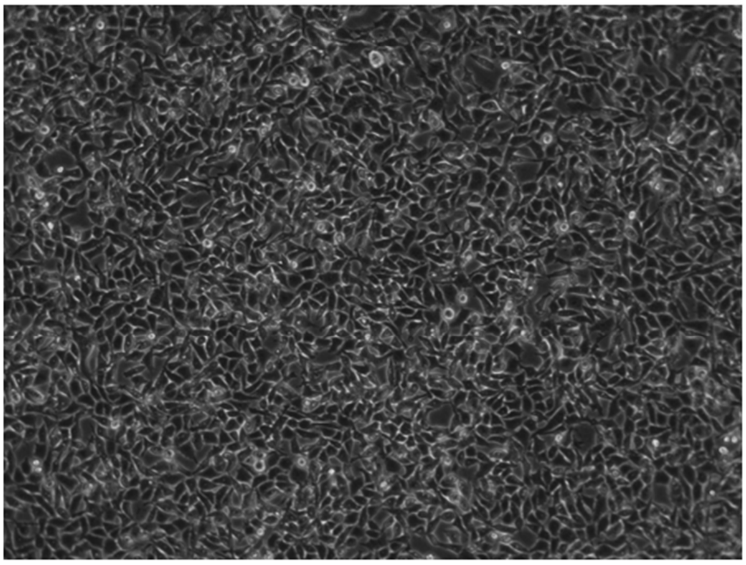

Figure S2: Optimization of the sensitive RKO cell seeding density for the collection of secretome prior to downstream proteomics analyses. The cells were seeded at three different densities as indicated and grown for 24 hours in serum-free medium. Cell viability was measured by the Trypan blue exclusion assay (A), and cell morphology was monitored by light microscopy at 10x magnification (B).

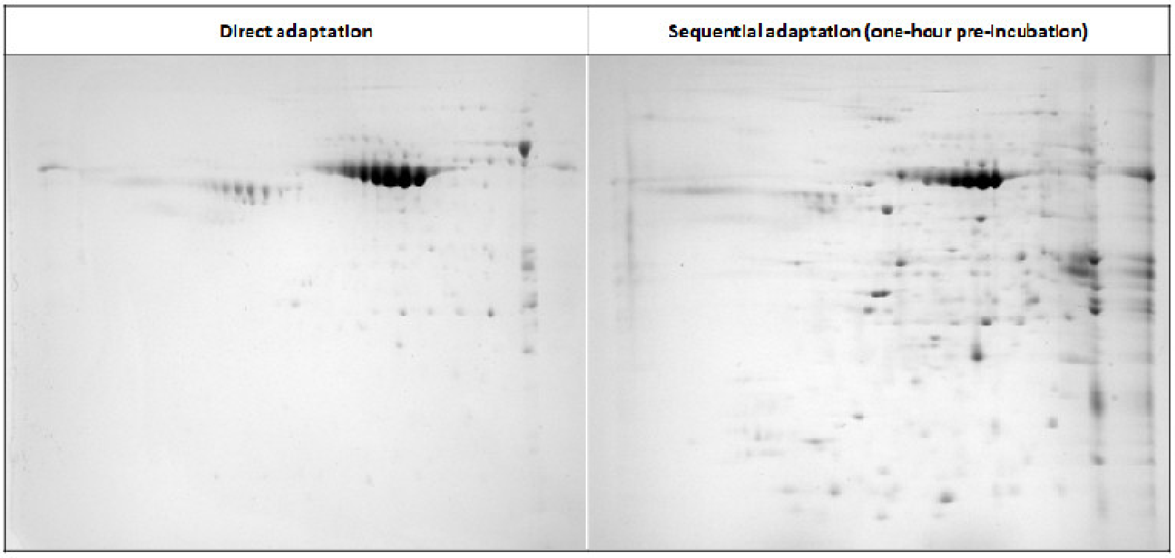

Figure S3: The effect of adaptation procedure to serum-free growth conditions on the secretome quality of the sensitive RKO cells. The cells were either directly switched from serum-containing medium into serum-free medium after extensive washing with PBS to remove serum and grown for 24 hours (direct adaptation) or subjected to one-hour pre-incubation in serum-free medium, the medium was discarded, and the cells were grown in fresh serum-free medium for 24 hours (sequential adaptation). The cell secretome was then analyzed by the means of two-dimensional gel electrophoresis (2-DE), and obtained gels were visually inspected.

2. PPI network and module analysis

2.1. Construction and modular analysis of the PPI network of upregulated proteins and identification of hub proteins

The PPI network (Figure S4A) consisted of 208 nodes and 128 edges (average node degree: 1.23; average local clustering coefficient: 0.344; PPI enrichment *p*-value: 8.66e-15). To find clusters in the PPI network, MCODE (https://apps.cytoscape.org/apps/mcode) was used to identify the most significant modules in the network by applying the following cut-off criteria: MCODE score ≥4, number of nodes ≥4, degree cutoff=2, node score cutoff=0.2, k-core=2 and maximum depth=100. Modular analysis revealed one significant module (Figure S4B) with MCODE score of 7.429 that consisted of 8 nodes and 26 edges. Cytoscape plugin cytoHubba (https://apps.cytoscape.org/apps/cytohubba) was employed to identify hub proteins in the network (Figure S4C). The hub proteins in the PPI network were ranked based on the Maximal Clique Centrality (MCC) values (Figure S4D). The proteins with highest MCC values were selected for further *in silico* validation analyses including MCM3 (DNA replication licensing factor MCM3), FEN1 (Flap endonuclease 1), MCM5 (DNA replication licensing factor MCM5), MCM2 (DNA Replication Licensing Factor MCM2), MCM6 (DNA Replication Licensing Factor MCM6), PCNA (Proliferating Cell Nuclear Antigen) and RPA1 (Replication protein A 70 kDa DNA-binding subunit).

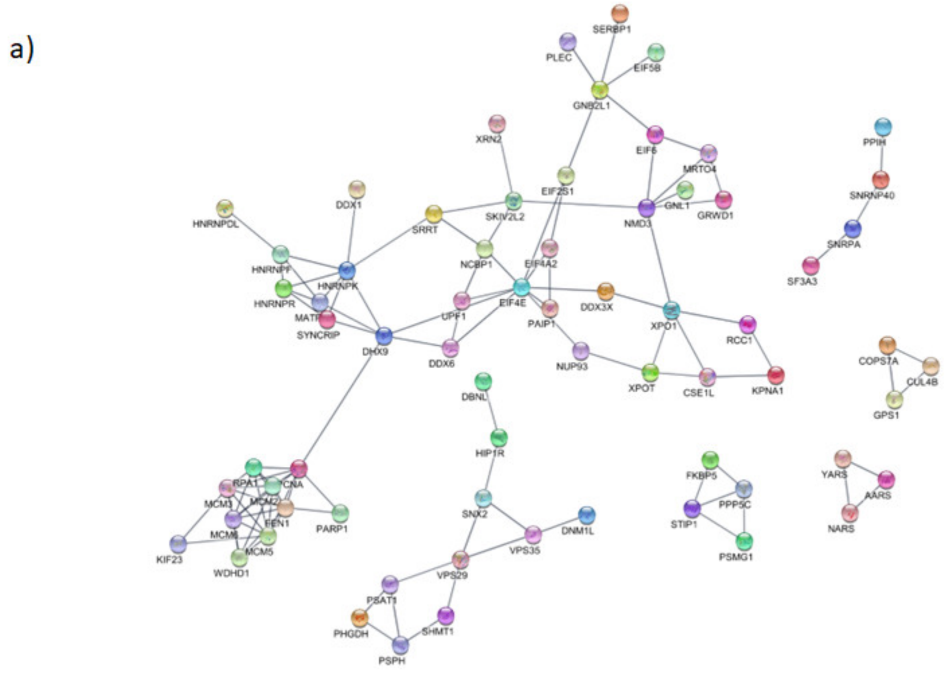

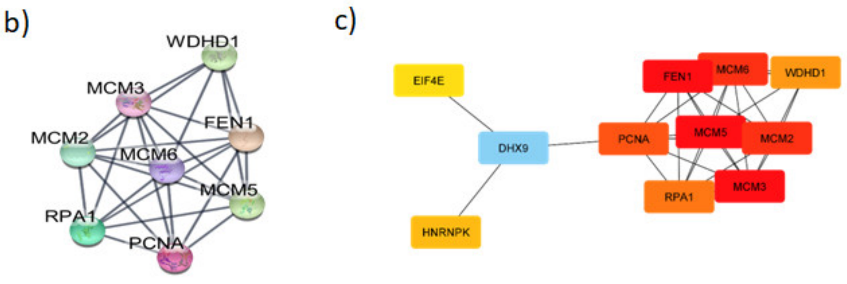

d)

Score	Name
842.0	MCM3
842.0	FEN1
842.0	MCM5
840.0	MCM2
840.0	MCM6
723.0	PCNA
720.0	RPA1
120.0	WDHD1
14.0	HNRNPK
12.0	EIF4E

FIGURE S4. Construction and modular analysis of the PPI network of upregulated proteins and identification of hub proteins. The PPI network was created by the online data-base STRING and visualized by the Cytoscape software (A). MCODE plugin of Cytoscape was used for modular analysis of the PPI network, where one significant module was identified (B). Cytoscape plugin cytoHubba was applied to identify hub proteins based on the maximal clique centrality (MCC) algorithm (C). Edges represent the protein-protein associations. Red nodes represent proteins with high MCC scores, whereas yellow nodes represent proteins with low MCC score (C). The proteins with high MCC scores were selected for further *in silico* analyses (D).

2.2. Construction and modular analysis of the PPI network of downregulated proteins and identification of hub proteins

Using STRING database and Cytoscape software, the PPI network of **downregulated proteins** was constructed (Figure S5A), which consisted of 87 nodes and 53 edges with an average node degree of 1.22 and an average local clustering coefficient of 0.329. The PPI enrichment p-value was $< 1.0\times10^{-16}$. MCODE analysis of the PPI network modules identified one significant module consisting of 6 nodes and 12 edges (MCODE score: 4.8) (Figures S5B and C). CytoHubba analysis revealed ten hub proteins in the significant module (Figure S5D), and among these, five were highly ranked according to the MCC score including HSPA5 (heat shock 70kDa protein 5 (glucose-regulated protein, 78kDa)), HSP90B1 (Heat Shock Protein 90 Beta Family Member 1), PDIA4 (Protein disulfide-isomerase A4), CALR (Calreticulin9 and ITGB1(Integrin beta-1).

a) b)

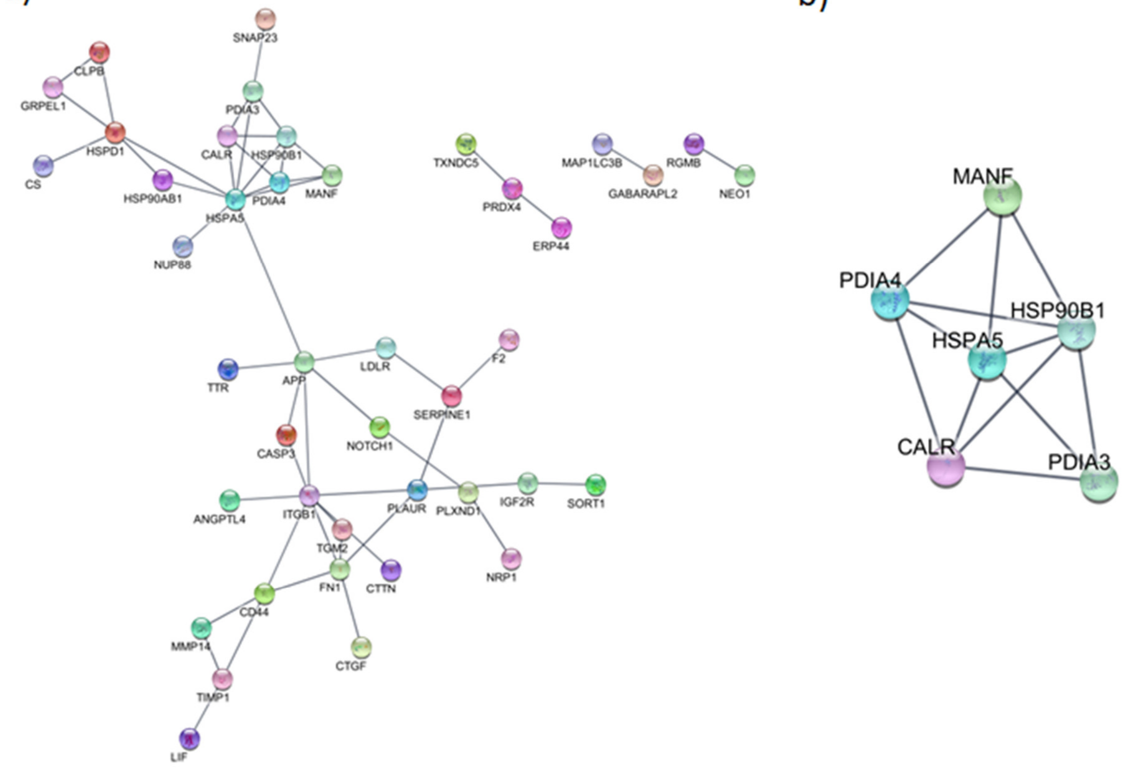

c)

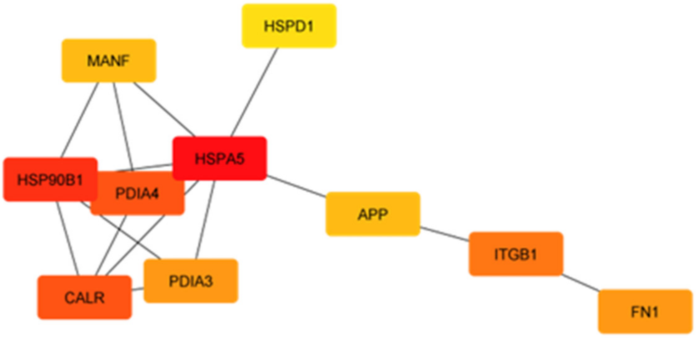

d)

Score	Name
22	HSPA5
18	HSP90B1
12	PDIA1
12	CALR
10	ITGB1
7	PDIA3
7	FN1
6	APP
6	MANF
5	HSPD1

FIGURE S5. Construction and modular analysis of the PPI network of **downregulated proteins** and identification of hub proteins. The PPI network was created by the online database STRING and visualized by the Cytoscape software (A). MCODE plugin of Cytoscape was used for modular analysis of the PPI network, where one significant module was identified (B). Cytoscape plugin cytoHubba was applied to identify hub proteins based on the maximal clique centrality (MCC) algorithm (C). Edges represent the protein-protein associations. Red nodes represent proteins with high MCC scores, whereas yellow nodes represent proteins with low MCC score (C). The proteins with high MCC scores were selected for further *in silico* analyses (D).

3. *In silico* evaluation of selected PPI network hub proteins in BRAF V600E-mutated colon cancer

We evaluated the expression of the genes encoding for the PPI hub proteins identified in our study in the database of tumor tissues from 48 colon cancer patients with BRAFV600E mutation *vs.* 478 tumor samples without BRAF mutation in the Cancer Genome Atlas (TCGA) dataset (Colorectal Adenocarcinoma, TCGA, PanCancer Atlas) using the cBioPortal for Cancer Genomics (https://www.cbioportal.org/).

3.1. In silico evaluation of selected hub proteins from the upregulated PPI network in BRAF V600E mutated colon cancer

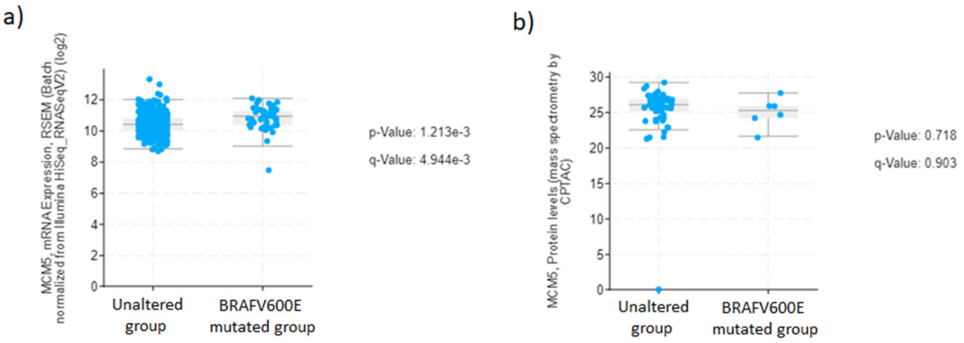

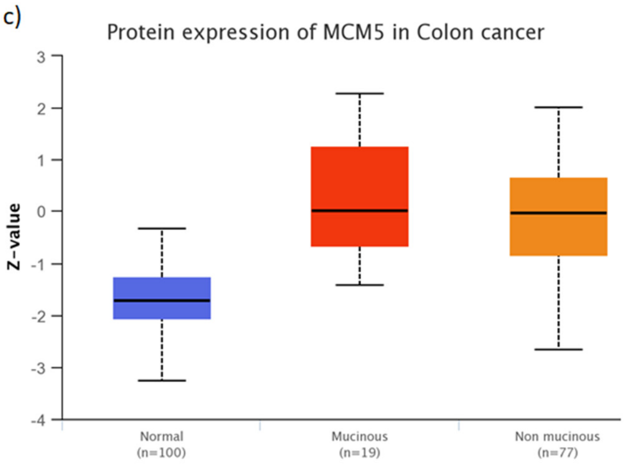

Comparison	Statistical significance
Normal *vs* Mucinous	4.089E-07
Normal *vs* Non mucinous	7.044E-25
Mucinous *vs* Non mucinous	2.149E-01

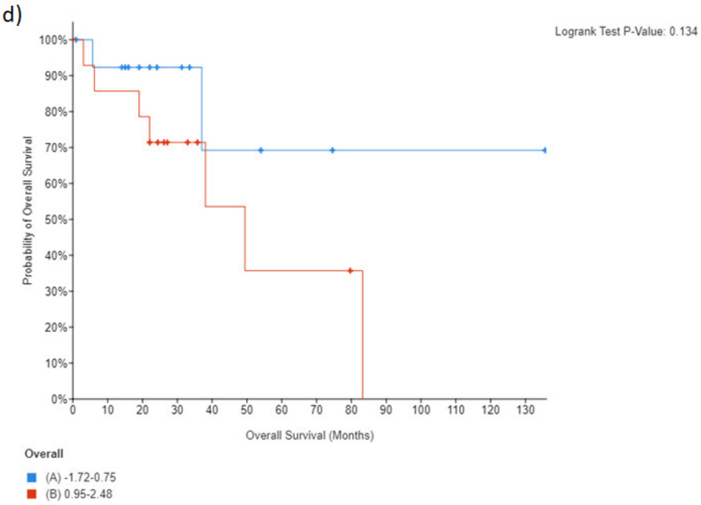

	Number of Cases, Total	Number of Events	Median Months Overall (95% CI)
(A) -1.72-0.75	15	2	NA
(B) 0.95-2.48	16	7	49.41 (38.07 - NA)

Figure S6. *In silico* validation and survival analysis of selected candidate protein MCM5 (minichromosome maintenance complex component 5) in BRAFV600E mutated colon cancer. Evaluation of the MCM5 expression at mRNA (A) and protein (B) level in the Colorectal Adenocarcinoma (TCGA, PanCancer Atlas) dataset using cBioPortal on-line tool. Expression analysis was conducted in 48 colon cancer samples with BRAFV600E mutation in comparison with 478 samples without BRAF mutation (unaltered group). The MCM5 protein expression was also analyzed in different histological types of colon cancer (normal, mucinous and non-mucinous) in the Clinical Proteomic Tumor Analysis Consortium (CPTAC) dataset using the UALCAN data analysis portal, where Z-values represent standard deviations from the median across samples for the given cancer type (C). Median overall survival analysis of MCM5 in 31 cases of colon adenocarcinoma in the TCGA PanCancer Atlas dataset using cBioPortal (D). The association between the MCM5 mRNA expression (mRNA expression z-scores relative to all samples (log RNA Seq V2 RSEM)) and the probability of overall survival is shown, where high- and low-mRNA expression groups of colon cancer patients were compared.

a)

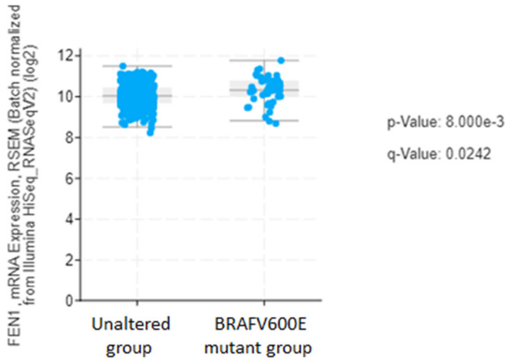

b)

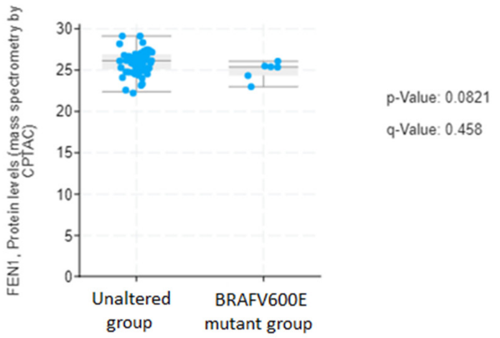

c)

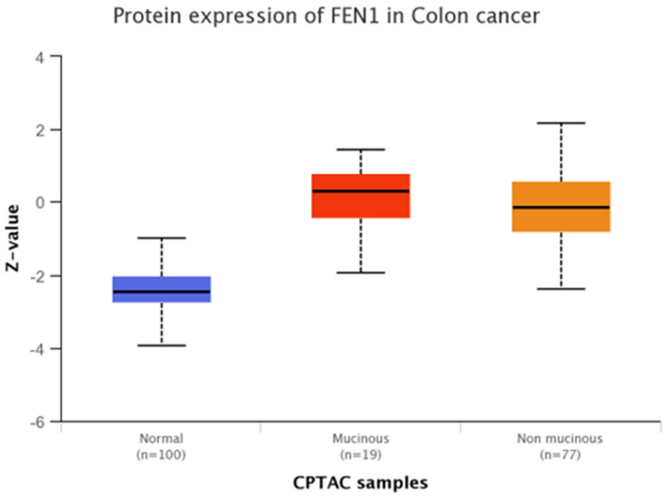

Comparison	Statistical significance
Normal *vs* Mucinous	3.140E-12
Normal *vs* Non mucinous	7.331E-35
Mucinous *vs* Non mucinous	2.280E-01

d)

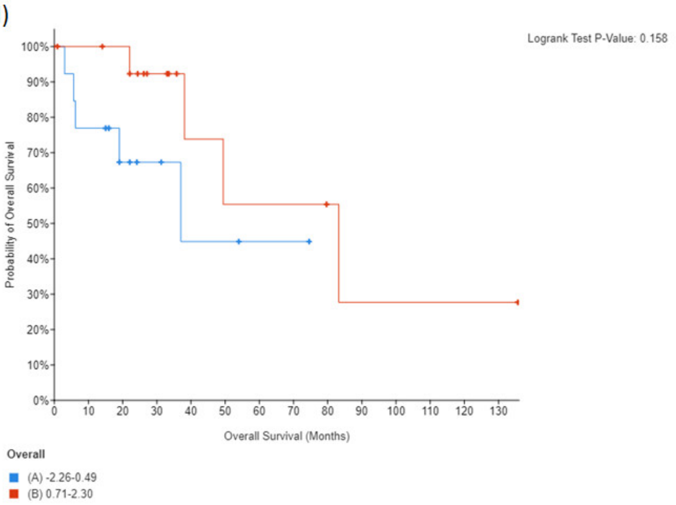

	Number of Cases, Total	Number of Events	Median Months Overall (95% CI)
(A) -2.26-0.49	15	5	37.02 (19.04 - NA)
(B) 0.71-2.30	16	4	83.24 (38.07 - NA)

Figure S7. *In silico* validation and survival analysis of selected candidate protein FEN1 (flap structure-specific endonuclease 1) in BRAFV600E mutated colon cancer. Evaluation of the FEN1 expression at mRNA (A) and protein (B) level in the Colorectal Adenocarcinoma (TCGA, PanCancer Atlas) dataset using cBioPortal on-line tool. Expression analysis was conducted in 48 colon cancer samples with BRAFV600E mutation in comparison with 478 samples without BRAF mutation (unaltered group). The FEN1 protein expression was also analyzed in different histological types of colon cancer (normal, mucinous and non-mucinous) in the Clinical Proteomic Tumor Analysis Consortium (CPTAC) dataset using the UALCAN data analysis portal, where Z-values represent standard deviations from the median across samples for the given cancer type (C). Median overall survival analysis of FEN1 in 31 cases of colon adenocarcinoma in the TCGA PanCancer Atlas dataset using cBioPortal (D). The association between the FEN1 mRNA expression (mRNA expression z-scores relative to all samples (log RNA Seq V2 RSEM)) and the probability of overall survival is shown, where high- and low-mRNA expression groups of colon cancer patients were compared.

3.2. In silico evaluation of selected hub proteins from the downregulated PPI network in BRAF V600E mutated colon cancer

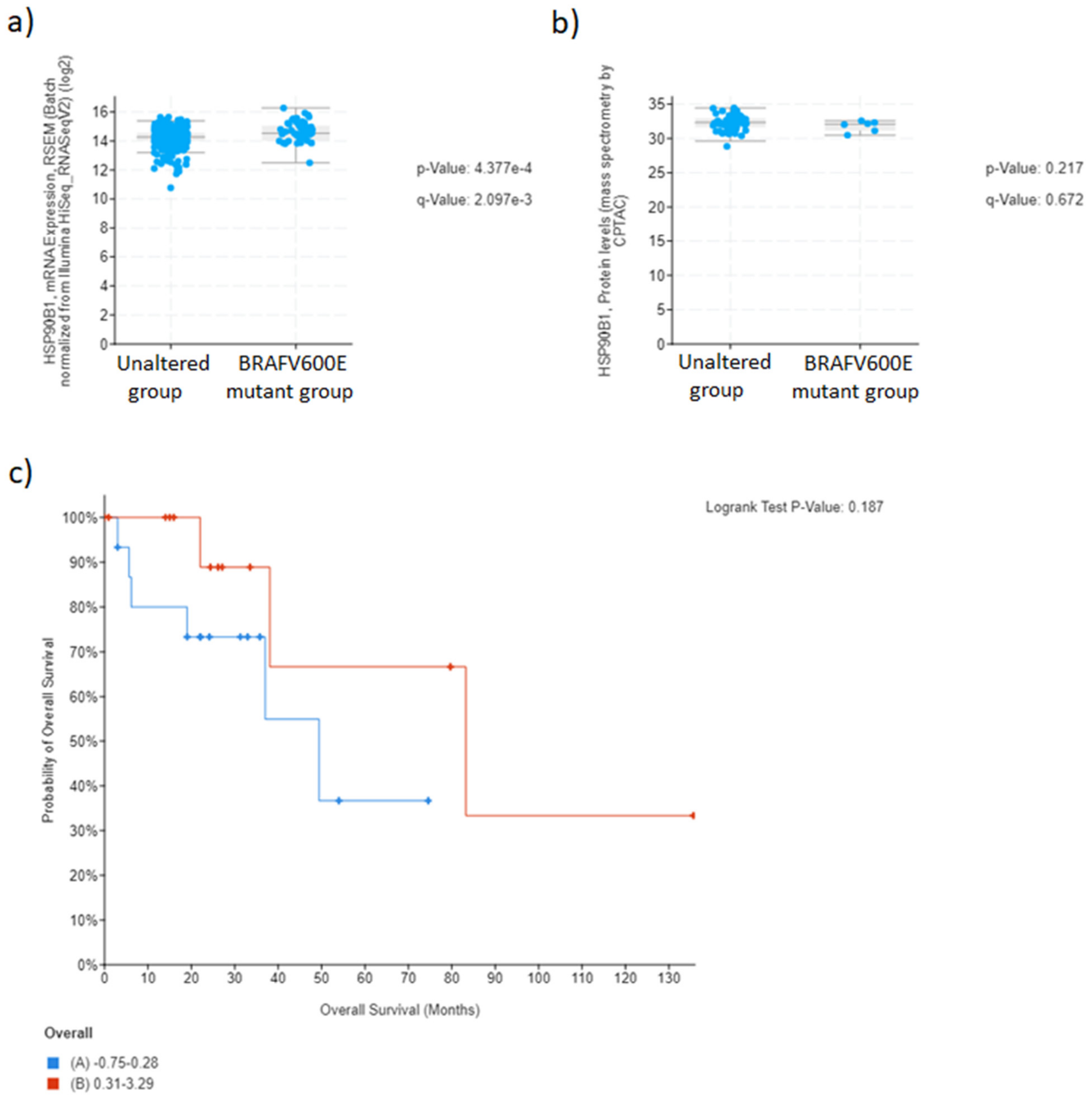

	Number of Cases, Total	Number of Events	Median Months Overall (95% CI)
(A) -0.75-0.28	15	6	49.41 (37.02 – NA)
(B) 0.31-3.29	16	3	83.24 (38.07 - NA)

Figure S8. *In silico* validation and survival analysis of selected candidate protein HSP90B1 (heat shock protein 90 beta family member 1) in BRAFV600E mutated colon cancer. Evaluation of the HSP90B1 expression at mRNA (A) and protein (B) level in the Colorectal Adenocarcinoma (TCGA, PanCancer Atlas)

dataset using cBioPortal on-line tool. Expression analysis was conducted in 48 colon cancer samples with BRAFV600E mutation in comparison with 478 samples without BRAF mutation (unaltered group). Median overall survival analysis of HSP90B1 in 31 cases of colon adenocarcinoma in the TCGA PanCancer Atlas dataset using cBioPortal (C). The association between the HSP90B1 mRNA expression (mRNA expression z-scores relative to all samples (log RNA Seq V2 RSEM)) and the probability of overall survival is shown, where high- and low-mRNA expression groups of colon cancer patients were compared.

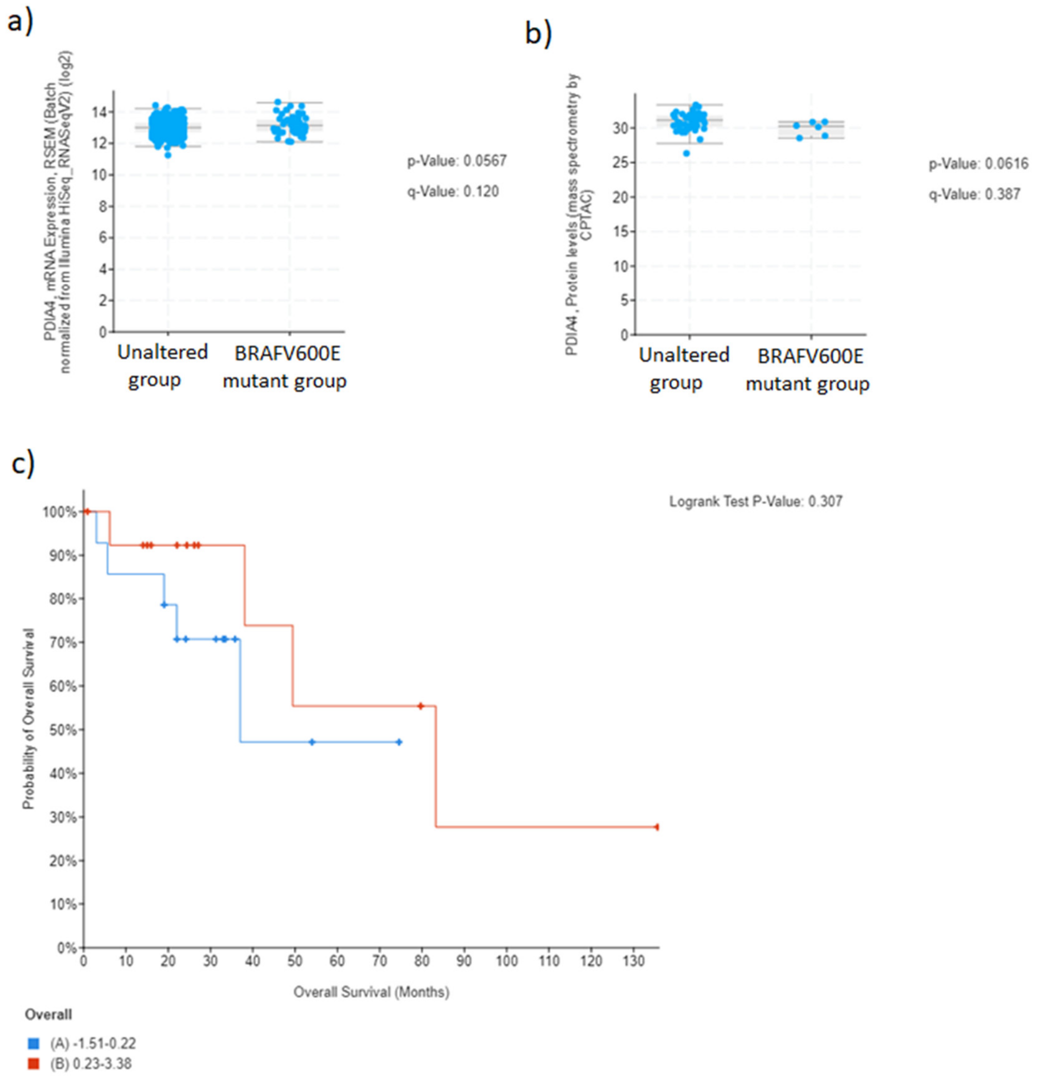

	Number of Cases, Total	Number of Events	Median Months Overall (95% CI)
(A) -1.51-0.22	15	5	37.02 (37.02 - NA)
(B) 0.23-3.38	16	4	83.24 (38.07 - NA)

Figure S9 *In silico* validation and survival analysis of selected candidate protein PDIA4 (protein disulfide isomerase family A member 4) in BRAFV600E mutated colon cancer. Evaluation of the PDIA4 expression at mRNA (A) and protein (B) level in the Colorectal Adenocarcinoma (TCGA, PanCancer Atlas) dataset using cBioPortal on-line tool. Expression analysis was conducted in 48 colon cancer samples with BRAFV600E mutation in comparison with 478 samples without BRAF mutation (unaltered group). Median overall survival analysis of PDIA4 in 31 cases of colon adenocarcinoma in the TCGA PanCancer Atlas dataset using cBioPortal (C). The association between the PDIA4 mRNA expression. The association between the RPA1 mRNA expression (mRNA expression z-scores relative to all samples (log RNA Seq V2 RSEM)) and the probability of overall survival is shown, where high- and low-mRNA expression groups of colon cancer patients were compared.

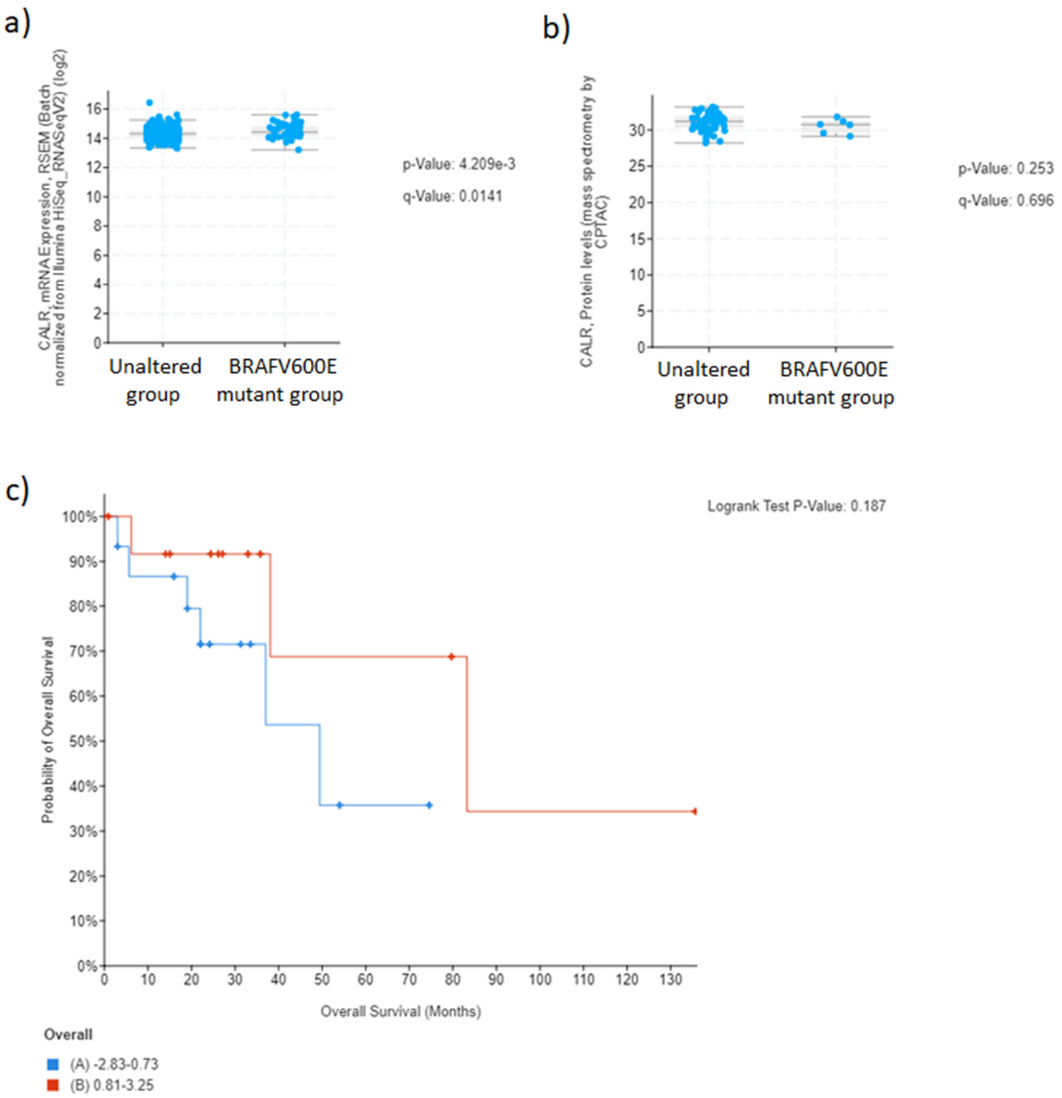

	Number of Cases, Total	Number of Events	Median Months Overall (95% CI)
(A) -2.83-0.73	15	6	49.41 (37.02 - NA)
(B) 0.81-3.25	16	3	83.24 (38.07 - NA)

Figure S10. *In silico* validation and survival analysis of selected candidate protein CALR (calreticulin) in BRAFV600E mutated colon cancer. Evaluation of the CALR expression at mRNA (A) and protein (B) level in the Colorectal Adenocarcinoma (TCGA, PanCancer Atlas) dataset using cBioPortal on-line tool. Expression analysis was conducted in 48 colon cancer samples with BRAFV600E mutation in comparison with 478 samples without BRAF mutation (unaltered group). Median overall survival analysis of CALR in 31 cases of colon adenocarcinoma in the TCGA PanCancer Atlas dataset using cBioPortal (C). The association between the CALR mRNA expression (mRNA expression z-scores relative to all samples (log RNA Seq V2 RSEM)) and the probability of overall survival is shown, where high- and low-mRNA expression groups of colon cancer patients were compared.

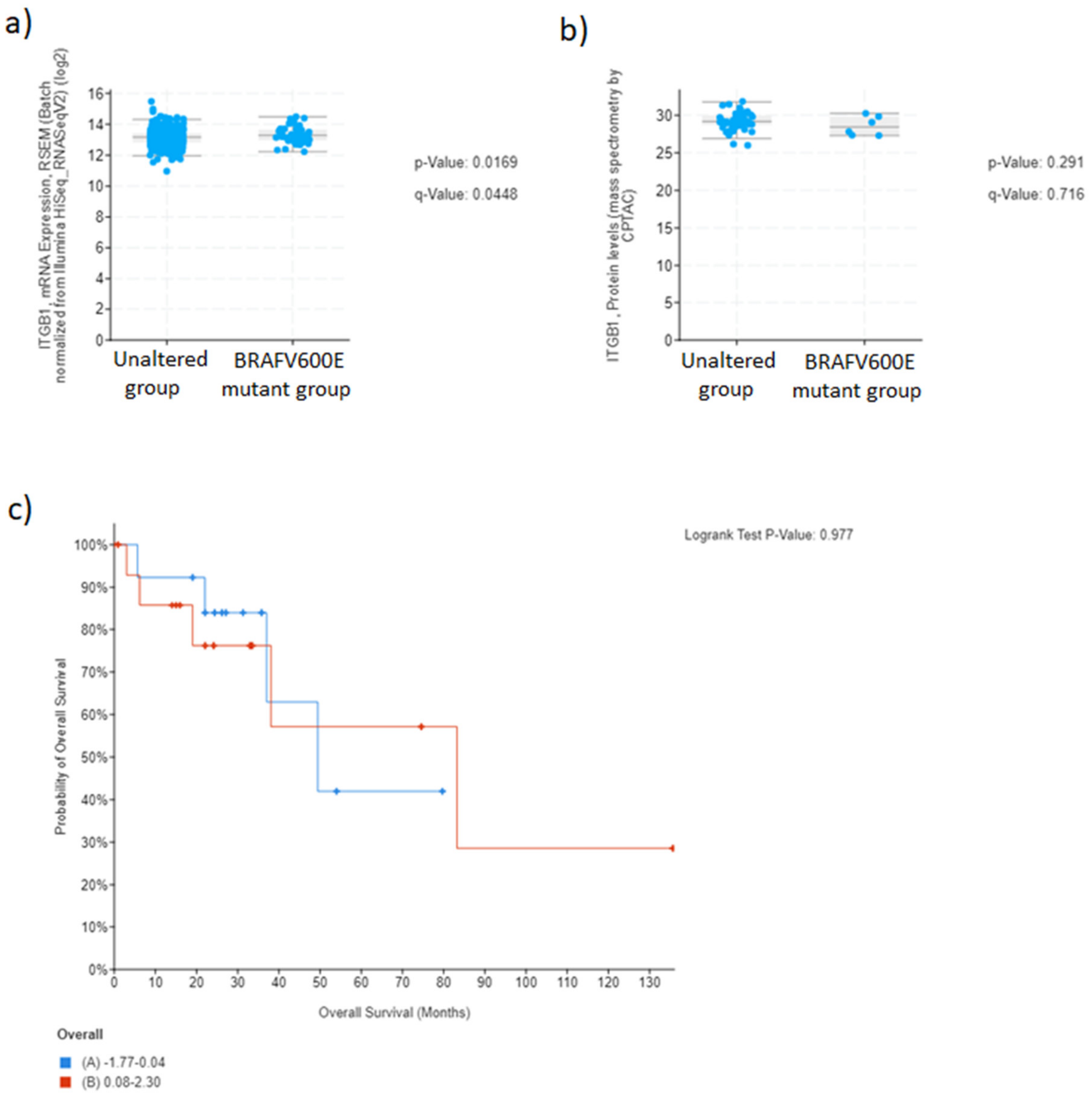

	Number of Cases, Total	Number of Events	Median Months Overall (95% CI)
(A) -1.77-0.04	15	4	49.41 (37.02 - NA)
(B) 0.08-2.30	16	5	83.24 (38.07 - NA)

Figure S11. *In silico* validation and survival analysis of selected candidate protein ITGB1 (integrin subunit beta 1) in BRAFV600E mutated colon cancer. Evaluation of the ITGB1 expression at mRNA (A) and protein (B) level in the Colorectal Adenocarcinoma (TCGA, PanCancer Atlas) dataset using cBioPortal on-line tool. Expression analysis was conducted in 48 colon cancer samples with BRAFV600E mutation in comparison with 478 samples without BRAF mutation (unaltered group). Median overall survival analysis of ITGB1 in 31 cases of colon adenocarcinoma in the TCGA PanCancer Atlas dataset using cBioPortal (C). The association between the ITGB1 mRNA expression (mRNA expression z-scores relative to all samples (log RNA Seq V2 RSEM)) and the probability of overall survival is shown, where high- and low-mRNA expression groups of colon cancer patients were compared.
